# Hexa-μ-acetato-chlorido­(μ-*N*,2-dioxodo­benzene-1-carboximidato)-μ_3_-oxido-tetra­iron(III)–water (1/1) and hexa-μ-acetato-(μ-*N*,2-dioxodo­benzene-1-carboximidato)fluorido-μ_3_-oxido-tri­pyridine­tetra­iron(III)–pyridine–water (1/1/0.24)

**DOI:** 10.1107/S2056989021009208

**Published:** 2021-09-14

**Authors:** Cassandra L. Ward, Matthew J. Allen, Jacob C. Lutter

**Affiliations:** a Lumingen Instrument Center, Wayne State University, 5101 Cass Avenue, Detroit, MI 48202, USA; b Department of Chemistry, Wayne State University, 5101 Cass Avenue, Detroit, MI 48202, USA

**Keywords:** crystal structure, iron(III), halide, hydroximate, acetate, μ_3_-oxo

## Abstract

The title compounds, [Fe_4_(C_2_H_3_O_2_)_6_(C_7_H_4_O_3_)FO(C_5_H_5_N)_3_]·C_5_H_5_N·0.24H_2_O (**1-F**) and [Fe_4_(C_2_H_3_O_2_)_6_(C_7_H_4_O_3_)ClO(C_5_H_5_N)_3_]·H_2_O (**1-Cl**) were synthesized using a self-assembly reaction in methanol and pyridine with stoichiometric addition of salicyl­hydroxamic acid (H_3_shi), acetic acid (HOAc), and the appropriate ferric halide salt. The compounds crystallize as solvates, where **1-F** has one pyridine mol­ecule that is disordered about a twofold axis and one water mol­ecule with an occupancy of 0.24 (2); and **1-Cl** has one water mol­ecule that is disordered over two sites with occupancies of 0.71 (1) and 0.29 (1). The space groups for each analog differ as **1-F** crystallizes in *Fdd*2 while **1-Cl** crystallizes in *P*2_1_. The difference in packing is due to changes in the inter­molecular inter­actions involving the different halides. The two mol­ecules are mostly isostructural, differing only by the torsion of the pyrine ligands and slight orientation changes in the acetate ligands. All of the iron(III) ions are in six-coordinate octa­hedral ligand field geometries but each one exhibits a unique coordination environment with various numbers of O (four to six) and N (nought to two) atom donors. Bond-valence sums confirm each iron is trivalent. The hydroximate ligand is bound to three iron(III) ions using a fused chelate motif similar to those in metallacrown compounds.

## Abstract 2

The title compounds Fe_4_(C_7_H_4_O_3_)O(C_2_H_3_O_2_)_6_(C_5_H_5_N)_3_ *X* where *X* is either Cl or F were synthesized using a self-assembly reaction in methanol and pyridine with stoicometric addition of salicyl­hydroxamic acid (H_3_shi), acetic acid (HOAc), and the appropriate ferric halide salt. The compound is remeniscent of hydroximate binding in metallacrown structures.

## Chemical context   

**Figure 1:**
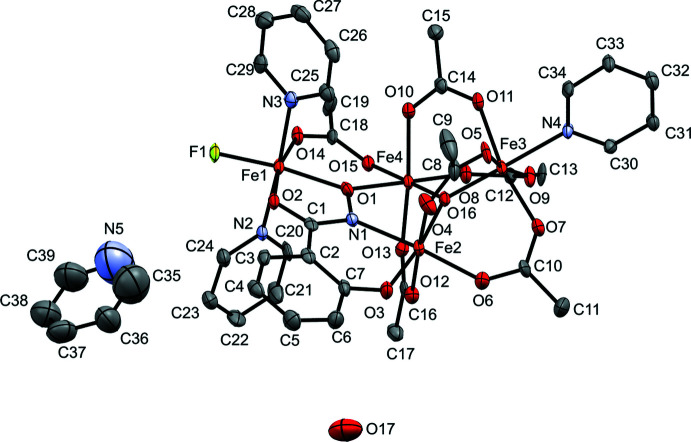
*ORTEP* representations from crystallographic data for **1-F**. Orange = iron, yellow = fluorine, light blue = nitro­gen, red = oxygen, gray = carbon. Displacement ellipsoids are drawn at the 50% probability level.

Examples of hydroximate binding as fused chelate rings has been dominated by a class of coordination compounds known as metallacrowns. First introduced by Pecoraro and Lah in 1989 (Pecoraro, 1989 ▸; Lah & Pecoraro, 1989 ▸), these compounds have since been tuned to explore many applications including host–guest binding, mol­ecular magnetism, and luminescence (Mezei *et al.*, 2007 ▸; Chow *et al.*, 2015 ▸; Lutter *et al.*, 2018 ▸). In particular, iron(III) 9-metallacrown-3 compounds have demonstrated inter­esting magnetocoolent properties (Chow *et al.*, 2016 ▸). Here, we describe two tetra-iron(III) compounds that have a fused chelate motif similar to metallacrowns but that are not examples of metallamacrocycles (Figs. 1 ▸ and 2 ▸). Instead, this fused chelate motif is complemented by six acetate ligands, a μ_3_-oxo ligand, and three pyridine ligands to complete the octa­hedral ligand fields of the four iron ions. These compounds were a serendipitous discovery from metallacrown synthesis that can be formed with their own rational self-assembly reaction.
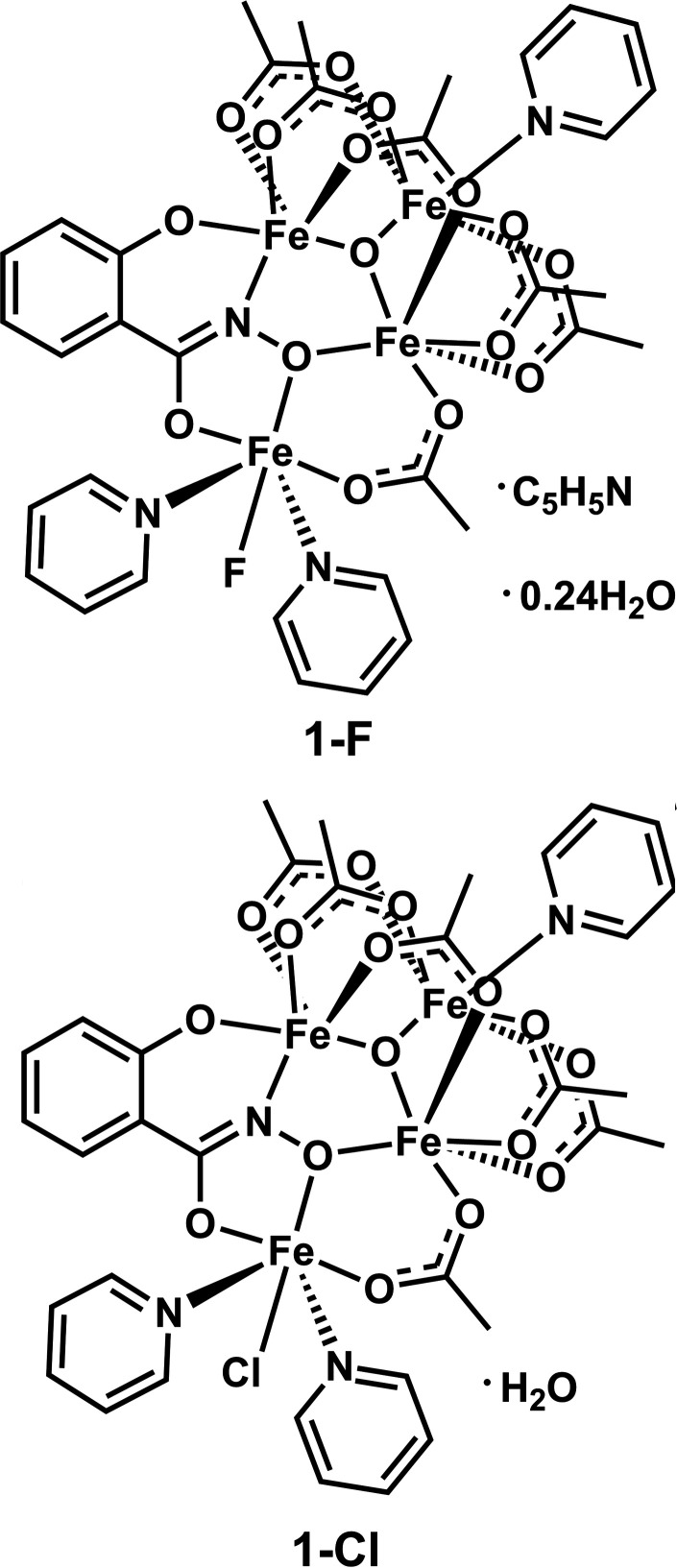


## Structural commentary   

**Figure 3:**
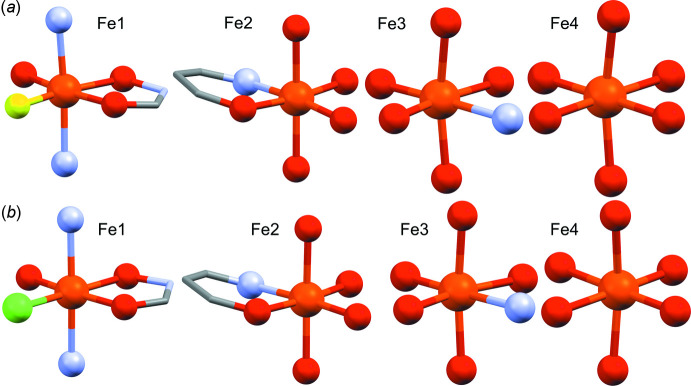
Depiction of iron(III) ion geometries from crystallographic data for (*a*) **1-F** and (*b*) **1-Cl**. Orange = iron, green = chlorine, yellow = fluorine, light blue = nitro­gen, red = oxygen, gray = carbon. Chelate rings from shi^3–^ are shown when appropriate.

**Figure 4:**
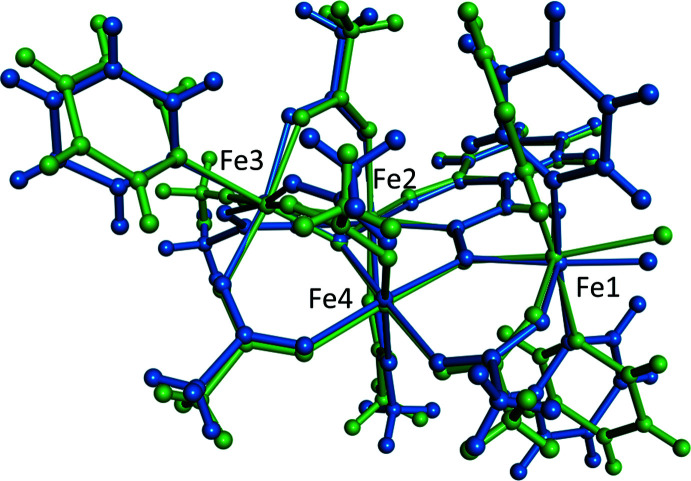
Overlay of **1-F** (blue) and **1-Cl** (green) shows near isomorphology between the two compounds.

Each of the iron(III) centers in **1-F** and **1-Cl** are in six-coord­inate octa­hedral ligand field geometries and bond-valence sums confirm that each iron ion is trivalent (Zheng *et al.*, 2017 ▸). More details are available in Tables 1 ▸ and 2 ▸. Fe1 is bound to the μ_2_-oxime oxygen and carbonyl oxygen of shi^3–^ to form a penta­gonal chelate ring, an oxygen from an acetate ligand, the nitro­gen from two pyridine ligands, and the respective halide for each compound. Fe2 is bound to the imino nitro­gen and phenolic oxygen of shi^3–^ to form a hexag­onal chelate ring, the μ_3_-oxo, and an oxygen from three acetate ligands. Fe3 is bound to the μ_3_-oxo, an oxygen from four acetate ligands, and the nitro­gen of a pyridine ligand. Fe4 is bound to the μ_3_-oxo, the μ_2_-oxime oxygen of shi^3–^, and an oxygen from four acetate ligands. Depictions of these coord­ination environments are shown in Fig. 3 ▸. Generally, for both compounds, the Fe—O_(oxo)_ bonds are shorter than the average, and the Fe—N_(pyridine)_ bonds are longer than the average (Tables 1 ▸ and 2 ▸). Geometric parameters, including bond lengths and angles for the coordination environment for the iron atoms, are given in Tables 3 ▸ and 4 ▸. An overlay of both structures shows some variation in pyridine and acetate ligand binding between **1-F** and **1-Cl** (Fig. 4 ▸). The acetates are close to uniform with only minor differences in binding orientation. The pyridine differ noticeably in torsion angle. The torsion angles of the two pyridines on Fe1 in **1-F** are 43.4 (5)° for C24—N2—Fe1—F1 and 149.3 (5)° for C25—N3—Fe1—F1, while the torsion angles of the two pyridines on Fe1 in **1-Cl** are 26.7 (5)° for C24—N2—Fe1—Cl1 and 104.5 (6)° for C25—N3—Fe1—Cl1. The torsion angle for the pyridine in **1-F** on Fe3 is 139.8 (5)° for C30—N4—Fe3—O5 while the torsion angle for the pyridine in **1-Cl** on Fe3 is 33.5 (4)° for C30—N4—Fe3—O5. These differences are likely due to the change in crystal packing between the two structures.

## Supra­molecular features   

Both compounds crystallize as solvates where **1-F** has one pyridine (N5 C35–39) and a 0.24 (2) occupancy water mol­ecule (O17), and **1-Cl** has one disordered water mol­ecule (O17 and O17*A*). The pyridine in **1-F** is disordered on a special position (twofold axis). This pyridine inter­acts with the main moiety *via* a hydrogen bond from an acetate C11—H11*A* bond to N5 on the pyridine. The pyridine also forms a hydrogen bond using using C39—H39 to donate to O6 from an acetate. The solvent water in **1-F** has two hydrogen bonds, where the O17—H17*E* bond donates to O13 on an acetate, and the C17—H17*A* bond on an acetate donates to O17. The solvent water in **1-Cl** is disordered over two sites with occupancies of 0.71 (1) and 0.29 (1) for the major and minor contributors. The major water site has three hydrogen bonds including: (i) the O17—H17*D* bond donating to Cl1, (ii) the O17—H17*E* bond donating to O3 in an acetate, and (iii) the C26—H26 bond of a pyridine donating to O17. The minor contributor has two hydrogen bonds, one where the C26—H26 bond in a pyridine donates to O17*A* and where the C6—H6 bond on shi^3–^ donates to O17*A*. Details of all hydrogen bonds, including distances and angles, are summarized in Tables 5 ▸ and 6 ▸.

The main moieties also have inter­molecular hydrogen-bonding inter­actions. In **1-F**, the C13—H13*A* bond on an acetate donates to F1, the C15—H15*B* bond on an acetate donates to F1, the C19—H19*B* bond on an acetate donates to F1, the C19—H19*C* bond of an acetate donates to O11 of an acetate, the C23—H23 bond on a pyridine donates to O5 of an acetate, the C26—H26 bond of a pyridine donates to O6 of an acetate, and the C31—H31 bond of a pyridine donates to O2 on shi^3–^. In **1-Cl**, the C15—H15*C* bond on an acetate donates to O15 from an acetate, the C19—H19*A* bond on an acetate donates to O11 from an acetate, the C21—H21 bond on a pyridine donates to O4 from an acetate, and the C23—H23 bond from a pyridine donates to Cl1. In addition to hydrogen bonding, **1-F** has π–π stacking between the pyridine containing N2 and C20—C24 and the pyridine containing N4 and C30—C34. There is no π–π stacking observed in **1-Cl**.

**Figure 5:**
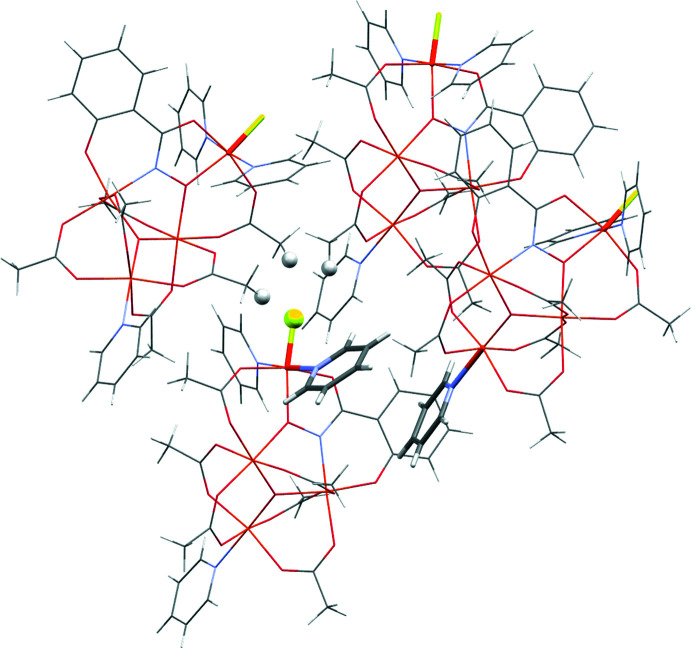
Representation of the crystal packing in **1-F** from crystallographic data. Hydrogen-bond pairs involving the halide are shown as spheres and the Fe—F bond is bolded for emphasis. Pyridines that have π–π stacking are bolded. Orange = iron, yellow = fluorine, light blue = nitro­gen, red = oxygen, gray = carbon.

**Figure 6:**
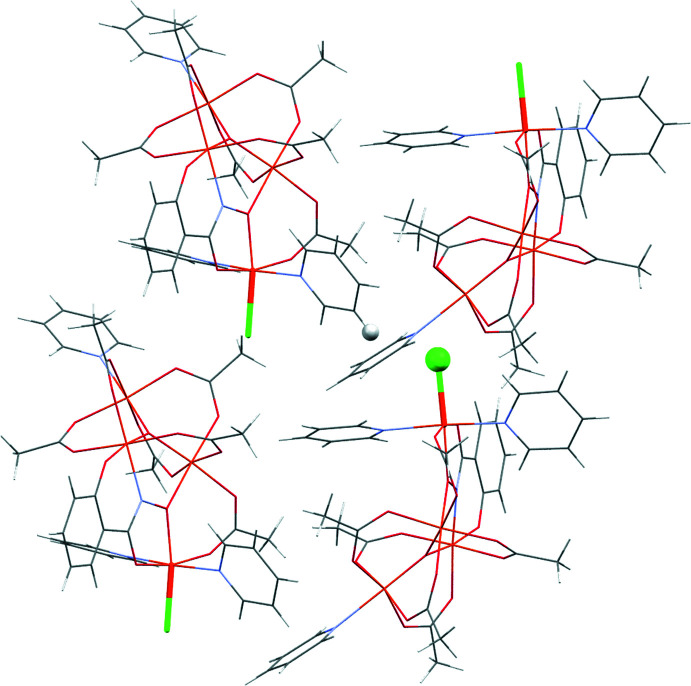
Representation of the crystal packing in **1-Cl** from crystallographic data. Hydrogen-bond pairs involving the halide are shown as spheres and the Fe–Cl bond is in bold for emphasis. Orange = iron, green = chlorine, light blue = nitro­gen, red = oxygen, gray = carbon.

Despite the fact that both compounds were synthesized using nearly identical procedures, each compound crystallizes in a unique space group where **1-F** is in *Fdd*2 and **1-Cl** is in *P*2_1_. The reason for the unique packing is likely due to the different chemistry of fluorine compared to chlorine. Fluorine has a smaller radius and is more electronegative than chlorine, and these properties have an effect on the overall packing of the compounds in their lattice. Essentially, the mol­ecules of **1-F** pack tighter than those of **1-Cl**. The main moiety inter­molecular hydrogen bonds discussed above demonstrate this difference. For **1-F**, there are seven inter­molecular hydrogen bonds where three of the hydrogen bonds involve fluorine (Fig. 5 ▸). However, in **1-Cl** there are four inter­molecular hydrogen bonds and only one of these hydrogen bonds involves the chlorine (Fig. 6 ▸). In addition, the difference in radius and electronegativity results in different lengths for hydrogen-bonding inter­actions, where **1-F** has proton-to-fluorine distances of 2.49, 2.54, and 2.60 Å while **1-Cl** has a proton-to-chlorine distance of 2.97 Å for their respective inter­molecular hydrogen bonds. Since the mol­ecules of **1-F** pack more tightly than those of **1-Cl**, their orientation is fixed such that all of the fluorine atoms of adjacent mol­ecules point towards the same direction of the unit cell and is enforced by π–π stacking of pyridine ligands (Fig. 5 ▸). In **1-Cl**, adjacent layers of mol­ecules point their chlorine atoms in opposite directions as there is less inter­action between the mol­ecules, likely due to pair-opposing mol­ecular dipoles (Fig. 6 ▸). This observation also suggests that **1-F** may have a crystallographic net dipole since all of the fluorines point in the same general direction.

## Database survey   

Two other compounds in the Cambridge Structural Database (Groom *et al.*, 2016 ▸) feature the same hydroximate coordination motif to three iron(III) ions shown in **1-F** and **1-Cl**, where both are iron(III) 9-met­alla­crown-3 compounds (Chow *et al.*, 2016 ▸): HADWOB and HADWUH. HADWOB is a 9-metallacrown-3 with three benzoate ligands that bridge the ring and central iron(III) ions and three methanol mol­ecules that are bound to ring iron(III) ions. HADWUH is a set of two 9-metallacrown-3 compounds with three isophthalate ligands that bridge the ring and central iron(III) ions as well as spanning the two rings into a dimeric structure. These structures are adaptations of another iron(III) 9-metallacrown-3 reported in 1989 (Lah *et al.*, 1989 ▸). The other major motif of a μ_3_-oxo combined with μ-acetato ligands on iron is not found in the Cambridge Structural Database.

## Synthesis and crystallization   

Fe_4_(shi)O(OAc)_6_(pyridine)_3_F (**1-F**): To a flask was added salicyl­hydroxamic acid (0.0766 g, 0.500 mmol, 1 equiv) and iron(III) fluoride trihydrate (0.3338 g, 2.000 mmol, 4 equiv). These solids were dissolved in a mixture of methanol (10 mL) and pyridine (2 mL), resulting in a dark-purple solution. Glacial acetic acid (0.200 mL, 3.50 mmol, 7 equiv) was added immediately, and the resulting solution was stirred for 1 h. The reaction mixture was gravity filtered using Whatman #2 filter paper, and the filtrate was allowed to evaporate slowly. After about one week, purple plates were obtained and diffracted. These plates were collected using vacuum filtration with #2 Whatman filter paper and a water aspirator and allowed to dry for 1 h before stopping the vacuum. Synthetic yield = 27% based on salicyl­hydroxamic acid. Elemental analysis of Fe_4_C_34_H_37_N_4_O_16_F (MM = 1000.06 g mol^−1^) observed (calculated): %C = 40.64 (40.83); %H = 3.83 (3.73); %N = 5.60 (5.95). Melting point = 530 K (decomposed). Selected FTIR peaks (ATR) in cm^−1^: 1585, 1560, 1535, 1496, 1408, 1329, 1263, 1221, 1149, 1097, 1070, 1041, 1015, 922, 862, 762, 696, 651, 636, 600, 544.

Fe_4_(shi)O(OAc)_6_(pyridine)_3_Cl (**1-Cl**): To a flask was added salicyl­hydroxamic acid (0.0383 g, 0.250 mmol, 1 equiv) and iron(III) chloride hexa­hydrate (0.2703 g, 1.000 mmol, 4 equiv). These solids were dissolved in a mixture of methanol (10 mL) and pyridine (2 mL), resulting in a dark-purple solution. Glacial acetic acid (0.100 mL, 1.75 mmol, 7 equiv) was added immediately, and the resulting solution was stirred for 1 h. The reaction mixture was gravity filtered using Whatman #2 filter paper, and the filtrate was allowed to evaporate slowly. After about one week, red–brown plates were observed and diffracted. These plates were collected using vacuum filtration with Whatman #2 filter paper and a water aspirator and allowed to dry for one h before stopping the vacuum. Synthetic yield = 59% based on salicyl­hydroxamic acid. Elemental analysis of Fe_4_C_34_H_37_N_4_O_16_Cl (MM = 1016.51 g mol^−1^) observed (calculated): %C = 40.22 (40.17); %H = 3.74 (3.67); %N = 5.77 (5.51). Melting point = 511 K (decomposed). Selected FTIR peaks (ATR) in cm^−1^: 1589, 1562, 1533, 1485, 1415, 1329, 1267, 1219, 1148, 1101, 1071, 1042, 1013, 930, 864, 766, 692, 633, 610, 565, 430.

Elemental analysis was performed by Midwest Microlabs.

## Refinement   

Crystal data, data collection and structure refinement details are summarized in Table 7 ▸. The absolute structure for both compounds were determined by refinement of the Flack parameter. For **1-F**, the pyridine containing N5 and C35–C39 is disordered around a special position (twofold axis) that was refined using a PART −1 command. The displacement parameters of these atoms were restrained with an esd of 0.01 using the ISOR command in *SHELXL* to limit excessive prolate character in displacement ellipsoids due to the disorder on a special position. A partial-occupancy water molecule containing O17 was refined to have an occupancy of 0.24 (2). Hydrogen atoms on O17 were located on the difference map and distances were restrained to 0.84 (2) Å for O–H bonds in water using a DFIX command in *SHELXL*. In addition, the distance between H—H atoms in the water mol­ecule was restrained to 1.35 (2) Å using a DANG command in *SHELXL*. These restraints maintain reasonable geometry for a water mol­ecule. Final refinement required an additional geometric constraint using the AFIX 3 command in *SHELXL* to stabilize the positions of these 0.24 (2) occupancy hydrogen atoms. For **1-Cl**, one disordered water molecule containing O17 was refined using a PART command and refined occupancies of 0.71 (1) and 0.29 (1). Hydrogen atoms for the water were found on the difference map and O—H bonds were restrained to 0.84 (2) Å using a DFIX command in *SHELXL*. The distance between H—H atoms in the water mol­ecule were restrained to 1.35 (2) Å using a DANG command in *SHELXL*. These restraints maintain reasonable geometry of the water mol­ecules.

## Supplementary Material

Crystal structure: contains datablock(s) 1-F, 1-Cl. DOI: 10.1107/S2056989021009208/zl5012sup1.cif

Structure factors: contains datablock(s) 1-F. DOI: 10.1107/S2056989021009208/zl50121-Fsup2.hkl

Structure factors: contains datablock(s) 1-Cl. DOI: 10.1107/S2056989021009208/zl50121-Clsup3.hkl

CCDC references: 2085824, 2085825

Additional supporting information:  crystallographic information; 3D view; checkCIF report

## Figures and Tables

**Figure 2:**
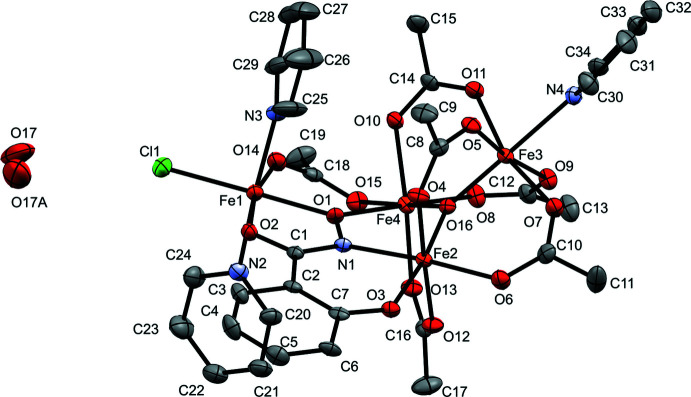
*ORTEP* representation from crystallographic data for **1-Cl**. Orange = iron, green = chlorine, light blue = nitro­gen, red = oxygen, gray = carbon. Displacement ellipsoids are drawn at the 50% probability level.

**Table 1:** Geometric information (Å) for **1-F**

Metal ID	Coordination number	Shape	Average bond length	Bond-valence sum^*a*^	Fe—O_(oxo)_bond length	Fe—N_(pyridine)_ bond length
Fe1	6	Octa­hedral	2.103	2.953	–	2.164, 2.194
Fe2	6	Octa­hedral	2.011	3.191	1.940	–
Fe3	6	Octa­hedral	2.026	3.116	1.866	2.207
Fe4	6	Octa­hedral	2.007	3.158	1.886	–

Note: (*a*) Zheng *et al.* (2017 ▸).

**Table 2:** Geometric information (Å) for **1-Cl**

Metal ID	Coordination number	Shape	Average bond length	Bond-valence sum^*a*^	Fe—O_(oxo)_bond length	Fe—N_(pyridine)_ bond length
Fe1	6	Octa­hedral	2.026	3.032	–	2.133, 2.171
Fe2	6	Octa­hedral	2.012	3.199	1.925	–
Fe3	6	Octa­hedral	2.020	3.149	1.871	2.169
Fe4	6	Octa­hedral	2.012	3.118	1.890	–

Note: (*a*) Zheng *et al.* (2017 ▸).

**Table 3:** Selected geometric parameters (Å, °) for 1-F[Chem scheme1]

Fe4—O16	1.890 (5)	Fe1—F1	1.843 (4)
Fe4—O15	2.016 (5)	Fe1—O2	1.978 (5)
Fe4—O13	2.021 (4)	Fe1—O14	1.987 (4)
Fe4—O10	2.023 (4)	Fe1—O1	2.043 (5)
Fe4—O8	2.034 (4)	Fe1—N3	2.133 (5)
Fe4—O1	2.056 (4)	Fe1—N2	2.171 (5)
Fe3—O16	1.872 (4)	Fe2—O3	1.902 (5)
Fe3—O9	1.997 (5)	Fe2—O16	1.925 (4)
Fe3—O5	2.020 (5)	Fe2—O6	2.021 (5)
Fe3—O11	2.024 (5)	Fe2—O12	2.057 (5)
Fe3—O7	2.037 (5)	Fe2—N1	2.083 (5)
Fe3—N4	2.170 (5)	Fe2—O4	2.084 (5)
			
O16—Fe4—O15	178.35 (19)	F1—Fe1—O2	93.63 (18)
O16—Fe4—O13	95.35 (18)	F1—Fe1—O14	97.73 (18)
O15—Fe4—O13	84.96 (18)	O2—Fe1—O14	168.62 (18)
O16—Fe4—O10	95.84 (18)	F1—Fe1—O1	170.62 (18)
O15—Fe4—O10	83.89 (18)	O2—Fe1—O1	77.18 (18)
O13—Fe4—O10	168.72 (19)	O14—Fe1—O1	91.44 (17)
O16—Fe4—O8	94.97 (18)	F1—Fe1—N3	89.35 (19)
O15—Fe4—O8	86.66 (18)	O2—Fe1—N3	93.10 (19)
O13—Fe4—O8	87.52 (18)	O14—Fe1—N3	87.6 (2)
O10—Fe4—O8	90.07 (18)	O1—Fe1—N3	93.0 (2)
O16—Fe4—O1	89.18 (18)	F1—Fe1—N2	87.15 (18)
O15—Fe4—O1	89.19 (18)	O2—Fe1—N2	88.72 (19)
O13—Fe4—O1	91.19 (18)	O14—Fe1—N2	91.2 (2)
O10—Fe4—O1	90.42 (18)	O1—Fe1—N2	90.74 (19)
O8—Fe4—O1	175.74 (18)	N3—Fe1—N2	176.2 (2)
O16—Fe3—O9	94.78 (18)	O3—Fe2—O16	171.98 (19)
O16—Fe3—O5	96.68 (18)	O3—Fe2—O6	92.3 (2)
O9—Fe3—O5	168.52 (18)	O16—Fe2—O6	95.12 (19)
O16—Fe3—O11	95.43 (18)	O3—Fe2—O12	91.36 (19)
O9—Fe3—O11	91.8 (2)	O16—Fe2—O12	92.38 (18)
O5—Fe3—O11	87.8 (2)	O6—Fe2—O12	85.01 (19)
O16—Fe3—O7	96.08 (19)	O3—Fe2—N1	86.4 (2)
O9—Fe3—O7	87.2 (2)	O16—Fe2—N1	86.6 (2)
O5—Fe3—O7	90.9 (2)	O6—Fe2—N1	174.8 (2)
O11—Fe3—O7	168.49 (18)	O12—Fe2—N1	90.0 (2)
O16—Fe3—N4	178.5 (2)	O3—Fe2—O4	85.9 (2)
O9—Fe3—N4	84.23 (19)	O16—Fe2—O4	90.72 (19)
O5—Fe3—N4	84.33 (18)	O6—Fe2—O4	92.3 (2)
O11—Fe3—N4	83.47 (19)	O12—Fe2—O4	176.1 (2)
O7—Fe3—N4	85.02 (19)	N1—Fe2—O4	92.6 (2)

**Table 4:** Selected geometric parameters (Å, °) for 1-Cl[Chem scheme1]

Cl1—Fe1	2.2963 (14)	Fe4—O15	2.039 (3)
Fe2—O3	1.918 (3)	Fe1—O2	1.957 (3)
Fe2—O16	1.940 (3)	Fe1—O14	1.982 (3)
Fe2—O6	2.015 (4)	Fe1—O1	2.025 (3)
Fe2—O12	2.050 (3)	Fe1—N3	2.165 (5)
Fe2—O4	2.070 (3)	Fe1—N2	2.194 (4)
Fe2—N1	2.075 (4)	Fe3—O16	1.866 (3)
Fe4—O16	1.886 (3)	Fe3—O5	2.012 (3)
Fe4—O13	2.011 (3)	Fe3—O11	2.016 (3)
Fe4—O1	2.033 (3)	Fe3—O9	2.023 (3)
Fe4—O10	2.035 (3)	Fe3—O7	2.030 (3)
Fe4—O8	2.039 (3)	Fe3—N4	2.206 (4)
			
O3—Fe2—O16	172.17 (14)	O2—Fe1—O14	167.24 (14)
O3—Fe2—O6	90.17 (14)	O2—Fe1—O1	77.46 (12)
O16—Fe2—O6	97.63 (13)	O14—Fe1—O1	90.32 (13)
O3—Fe2—O12	90.00 (14)	O2—Fe1—N3	94.22 (15)
O16—Fe2—O12	91.14 (14)	O14—Fe1—N3	88.66 (16)
O6—Fe2—O12	86.91 (14)	O1—Fe1—N3	86.72 (15)
O3—Fe2—O4	88.54 (14)	O2—Fe1—N2	89.67 (15)
O16—Fe2—O4	90.71 (13)	O14—Fe1—N2	86.85 (16)
O6—Fe2—O4	90.10 (14)	O1—Fe1—N2	90.99 (15)
O12—Fe2—O4	176.67 (15)	N3—Fe1—N2	174.96 (17)
O3—Fe2—N1	85.84 (14)	O2—Fe1—Cl1	93.87 (10)
O16—Fe2—N1	86.39 (14)	O14—Fe1—Cl1	98.56 (11)
O6—Fe2—N1	175.53 (15)	O1—Fe1—Cl1	170.45 (10)
O12—Fe2—N1	91.08 (15)	N3—Fe1—Cl1	89.97 (12)
O4—Fe2—N1	91.80 (15)	N2—Fe1—Cl1	92.98 (12)
O16—Fe4—O13	95.04 (14)	O16—Fe3—O5	93.99 (14)
O16—Fe4—O1	88.71 (13)	O16—Fe3—O11	98.53 (13)
O13—Fe4—O1	91.05 (14)	O5—Fe3—O11	89.69 (14)
O16—Fe4—O10	96.37 (13)	O16—Fe3—O9	93.46 (14)
O13—Fe4—O10	168.28 (14)	O5—Fe3—O9	172.54 (14)
O1—Fe4—O10	91.88 (13)	O11—Fe3—O9	88.91 (14)
O16—Fe4—O8	93.00 (13)	O16—Fe3—O7	97.10 (14)
O13—Fe4—O8	87.50 (14)	O5—Fe3—O7	89.76 (14)
O1—Fe4—O8	177.85 (14)	O11—Fe3—O7	164.36 (14)
O10—Fe4—O8	89.23 (14)	O9—Fe3—O7	89.61 (14)
O16—Fe4—O15	176.24 (14)	O16—Fe3—N4	178.69 (15)
O13—Fe4—O15	86.51 (14)	O5—Fe3—N4	84.80 (15)
O1—Fe4—O15	87.84 (14)	O11—Fe3—N4	81.96 (14)
O10—Fe4—O15	82.26 (13)	O9—Fe3—N4	87.76 (15)
O8—Fe4—O15	90.48 (14)	O7—Fe3—N4	82.43 (15)

**Table 5:** Hydrogen-bond geometry (Å, °) for 1-F[Chem scheme1]

*D*—H⋯*A*	*D*—H	H⋯*A*	*D*⋯*A*	*D*—H⋯*A*
C11—H11*A*⋯N5^i^	0.98	2.37	3.23 (3)	146
C13—H13*A*⋯F1^ii^	0.98	2.54	3.389 (7)	145
C15—H15*B*⋯F1^iii^	0.98	2.49	3.454 (8)	168
C17—H17*A*⋯O17^iv^	0.98	2.60	3.42 (3)	142
C19—H19*B*⋯F1^iii^	0.98	2.60	3.520 (9)	156
C19—H19*C*⋯O11^v^	0.98	2.47	3.446 (8)	172
C20—H20⋯O13	0.95	2.64	3.269 (8)	124
C20—H20⋯O15	0.95	2.49	3.365 (8)	153
C23—H23⋯O5^i^	0.95	2.55	3.473 (9)	164
C25—H25⋯O1	0.95	2.60	3.149 (8)	117
C26—H26⋯O6^vi^	0.95	2.64	3.431 (8)	141
C29—H29⋯F1	0.95	2.46	2.930 (8)	110
C31—H31⋯O2^ii^	0.95	2.48	3.282 (8)	143
C39—H39⋯O6^vii^	0.95	2.54	3.27 (2)	134
O17—H17*E*⋯O13^viii^	0.84	2.15	2.97 (3)	164

Symmetry codes: (i) x+{\script{1\over 4}}, -y+{\script{3\over 4}}, z+{\script{3\over 4}}; (ii) x, y, z-1; (iii) -x+1, -y+{\script{1\over 2}}, z-{\script{1\over 2}}; (iv) -x+{\script{3\over 4}}, y+{\script{1\over 4}}, z+{\script{3\over 4}}; (v) -x+1, -y+{\script{1\over 2}}, z+{\script{1\over 2}}; (vi) x-{\script{1\over 4}}, -y+{\script{3\over 4}}, z+{\script{1\over 4}}; (vii) x-{\script{1\over 4}}, -y+{\script{3\over 4}}, z-{\script{3\over 4}}; (viii) -x+{\script{3\over 4}}, y-{\script{1\over 4}}, z-{\script{3\over 4}}.

**Table 6:** Hydrogen-bond geometry (Å, °) for 1-Cl[Chem scheme1]

*D*—H⋯*A*	*D*—H	H⋯*A*	*D*⋯*A*	*D*—H⋯*A*
C6—H6⋯O17*A* ^i^	0.95	2.64	3.486 (19)	149
C15—H15*C*⋯O15^ii^	0.98	2.62	3.581 (7)	166
C19—H19*A*⋯O11^iii^	0.98	2.61	3.424 (7)	141
C20—H20⋯N1	0.95	2.65	3.429 (7)	140
C20—H20⋯O1	0.95	2.53	3.100 (6)	118
C21—H21⋯O4^iv^	0.95	2.56	3.403 (6)	148
C23—H23⋯Cl1^v^	0.95	2.97	3.697 (6)	135
C24—H24⋯Cl1	0.95	2.75	3.301 (6)	118
C25—H25⋯O2	0.95	2.55	3.126 (7)	119
C26—H26⋯O17^ii^	0.95	2.20	3.014 (12)	144
C26—H26⋯O17*A* ^ii^	0.95	2.60	3.17 (3)	118
C29—H29⋯O14	0.95	2.48	3.007 (7)	115
O17—H17*D*⋯Cl1^vi^	0.85 (3)	2.80 (3)	3.647 (9)	174 (15)
O17—H17*E*⋯O3^vii^	0.84 (3)	2.03 (5)	2.830 (8)	159 (12)

Symmetry codes: (i) x, y, z+1; (ii) -x, y+{\script{1\over 2}}, -z+1; (iii) -x, y-{\script{1\over 2}}, -z+1; (iv) -x, y-{\script{1\over 2}}, -z+2; (v) -x-1, y-{\script{1\over 2}}, -z+1; (vi) x+1, y, z; (vii) x, y, z-1.

**Table 7:** Experimental details

	**1-F**	**1-Cl**
Crystal data		
Chemical formula	[Fe_4_(C_2_H_3_O_2_)_6_(C_7_H_4_O_3_)FO(C_5_H_5_N)_5_]·C_5_H_5_N·0.24H_2_O	[Fe_4_(C_2_H_3_O_2_)_6_(C_7_H_4_O_3_)ClO(C_5_H_5_N)_3_]·H_2_O
*M*_r_	2087.76	1034.54
Crystal system, space group	Orthorhombic, *F* *d* *d*2	Monoclinic, *P*2_1_
Temperature (K)	100	100
*a*, *b*, *c* (Å)	31.676 (2), 44.806 (3), 12.6056 (8)	11.8460 (6), 15.5041 (7), 12.6425 (6)
α, β, γ (°)	90, 90, 90	90, 115.449 (1), 90
*V* (Å^3^)	17891 (2)	2096.64 (17)
*Z*	8	2
Radiation type	Mo *K*α	Mo *K*α
μ (mm^−1^)	1.35	1.50
Crystal size (mm)	0.33 × 0.30 × 0.14	0.28 × 0.28 × 0.14

Data collection		
Diffractometer	Bruker APEXII CCD	Bruker APEXII CCD
Absorption correction	Multi-scan (*SADABS*; Krause *et al.*, 2015 ▸)	Multi-scan (*SADABS*; Krause *et al.*, 2015 ▸)
*T*_min_, *T*_max_	0.670, 0.745	0.688, 0.745
No. of measured, independent and observed [*I* > 2σ(*I*)] reflections	102006, 8265, 7012	52843, 8621, 7877
*R*_int_	0.098	0.048
(sin θ/λ)_max_ (Å^−1^)	0.604	0.627

Refinement		
*R*[*F*^2^ > 2σ(*F* ^2^)], *wR*(*F* ^2^), *S*	0.039, 0.090, 1.05	0.033, 0.071, 1.06
No. of reflections	8265	8621
No. of parameters	604	570
No. of restraints	58	7
H-atom treatment	H-atom parameters constrained	H atoms treated by a mixture of independent and constrained refinement
Δρ_max_, Δρ_min_ (e Å^−3^)	0.47, −0.38	0.43, −0.34
Absolute structure	*Via* refinement	*Via* refinement
Absolute structure parameter	0.05 (2)	0.014 (16)

Computer programs: *APEX2* (Bruker, 2004 ▸), *SAINT* (Bruker, 2001 ▸), *SHELXT2018/3* (Sheldrick, 2015*a* ▸), *SHELXL2018/3* (Sheldrick, 2015*b* ▸), and *SHELXTL* (Sheldrick, 2008 ▸).

## supplementary crystallographic information

### Hexa-µ-acetato-(µ-N,2-dioxodobenzene-1-carboximidato)fluorido-µ3-oxido-tripyridinetetrairon(III)–pyridine–water (1/1/0.24) (1-F) Crystal data

**Table:**

[Fe_4_(C_2_H_3_O_2_)_6_(C_7_H_4_O_3_)FO(C_5_H_5_N)_5_]·C_5_H_5_N·0.24H_2_O	*D*_x_ = 1.550 Mg m^−^^3^
*M**_r_* = 2087.76	Melting point: 257 K
Orthorhombic, *F**d**d*2	Mo *K*α radiation, λ = 0.71073 Å
*a* = 31.676 (2) Å	Cell parameters from 9974 reflections
*b* = 44.806 (3) Å	θ = 2.2–25.1°
*c* = 12.6056 (8) Å	µ = 1.35 mm^−^^1^
*V* = 17891 (2) Å^3^	*T* = 100 K
*Z* = 8	Plate, purple
*F*(000) = 8534	0.33 × 0.30 × 0.14 mm

### Hexa-µ-acetato-(µ-N,2-dioxodobenzene-1-carboximidato)fluorido-µ3-oxido-tripyridinetetrairon(III)–pyridine–water (1/1/0.24) (1-F) Data collection

**Table:**

Bruker APEXII CCD diffractometer	7012 reflections with *I* > 2σ(*I*)
Radiation source: sealed tube	*R*_int_ = 0.098
φ and ω scans	θ_max_ = 25.4°, θ_min_ = 1.6°
Absorption correction: multi-scan (SADABS; Krause *et al.*, 2015)	*h* = −38→38
*T*_min_ = 0.670, *T*_max_ = 0.745	*k* = −54→54
102006 measured reflections	*l* = −15→15
8265 independent reflections	

### Hexa-µ-acetato-(µ-N,2-dioxodobenzene-1-carboximidato)fluorido-µ3-oxido-tripyridinetetrairon(III)–pyridine–water (1/1/0.24) (1-F) Refinement

**Table:**

Refinement on *F*^2^	Hydrogen site location: mixed
Least-squares matrix: full	H-atom parameters constrained
*R*[*F*^2^ > 2σ(*F*^2^)] = 0.039	*w* = 1/[σ^2^(*F*_o_^2^) + (0.0392*P*)^2^ + 78.008*P*] where *P* = (*F*_o_^2^ + 2*F*_c_^2^)/3
*wR*(*F*^2^) = 0.090	(Δ/σ)_max_ = 0.001
*S* = 1.05	Δρ_max_ = 0.47 e Å^−^^3^
8265 reflections	Δρ_min_ = −0.38 e Å^−^^3^
604 parameters	Extinction correction: SHELXL-2018/3 (Sheldrick 2018), Fc^*^=kFc[1+0.001xFc^2^λ^3^/sin(2θ)]^-1/4^
58 restraints	Extinction coefficient: 0.000039 (8)
Primary atom site location: dual	Absolute structure: Refined as an inversion twin
Secondary atom site location: difference Fourier map	Absolute structure parameter: 0.05 (2)

### Hexa-µ-acetato-(µ-N,2-dioxodobenzene-1-carboximidato)fluorido-µ3-oxido-tripyridinetetrairon(III)–pyridine–water (1/1/0.24) (1-F) Special details

**Table:**

Geometry. All esds (except the esd in the dihedral angle between two l.s. planes) are estimated using the full covariance matrix. The cell esds are taken into account individually in the estimation of esds in distances, angles and torsion angles; correlations between esds in cell parameters are only used when they are defined by crystal symmetry. An approximate (isotropic) treatment of cell esds is used for estimating esds involving l.s. planes.
Refinement. Refined as a two-component inversion twin. A lattice pyridine containing N5 and C35 through C39 lies on a twofold axis and was refined using a PART -1 command. The partial occupancy of lattice water O17 was refined using a free variable, and the H atoms were found on the difference map and refined with DFIX and DANG commands.

### Hexa-µ-acetato-(µ-N,2-dioxodobenzene-1-carboximidato)fluorido-µ3-oxido-tripyridinetetrairon(III)–pyridine–water (1/1/0.24) (1-F) Fractional atomic coordinates and isotropic or equivalent isotropic displacement parameters (Å^2^)

**Table:**

	*x*	*y*	*z*	*U*_iso_*/*U*_eq_	Occ. (<1)
Fe4	0.54162 (3)	0.31181 (2)	0.77891 (6)	0.0170 (2)	
Fe3	0.48707 (3)	0.34874 (2)	0.60174 (7)	0.0172 (2)	
Fe1	0.52740 (3)	0.30748 (2)	1.07300 (7)	0.0178 (2)	
Fe2	0.53647 (3)	0.38430 (2)	0.79611 (7)	0.0189 (2)	
C1	0.52500 (18)	0.36899 (15)	1.0345 (5)	0.0177 (14)	
C2	0.52547 (19)	0.40090 (15)	1.0584 (5)	0.0178 (14)	
C3	0.5175 (2)	0.40924 (16)	1.1646 (5)	0.0200 (15)	
H3	0.509743	0.394293	1.214328	0.024*	
C4	0.5208 (2)	0.43821 (16)	1.1980 (5)	0.0252 (17)	
H4	0.515227	0.443221	1.269894	0.030*	
C5	0.5322 (2)	0.46024 (18)	1.1260 (6)	0.0302 (18)	
H5	0.534580	0.480378	1.148789	0.036*	
C6	0.5401 (2)	0.45296 (16)	1.0213 (6)	0.0265 (16)	
H6	0.547500	0.468274	0.972619	0.032*	
C7	0.5372 (2)	0.42337 (16)	0.9856 (5)	0.0203 (15)	
C8	0.4415 (2)	0.39185 (17)	0.7444 (5)	0.0284 (17)	
C9	0.3996 (3)	0.4050 (2)	0.7751 (7)	0.053 (3)	
H9A	0.404201	0.422996	0.817643	0.080*	
H9B	0.383642	0.390370	0.816831	0.080*	
H9C	0.383705	0.410109	0.711027	0.080*	
C10	0.5404 (2)	0.40351 (17)	0.5665 (5)	0.0278 (17)	
C11	0.5638 (3)	0.4222 (2)	0.4871 (7)	0.055 (3)	
H11A	0.554177	0.442903	0.491977	0.083*	
H11B	0.558316	0.414546	0.415493	0.083*	
H11C	0.594135	0.421223	0.501821	0.083*	
C12	0.5479 (2)	0.30150 (15)	0.5414 (5)	0.0197 (14)	
C13	0.5673 (2)	0.28292 (16)	0.4548 (5)	0.0250 (16)	
H13A	0.564363	0.293303	0.386799	0.038*	
H13B	0.552940	0.263589	0.451135	0.038*	
H13C	0.597333	0.279782	0.470129	0.038*	
C14	0.4545 (2)	0.29360 (15)	0.7173 (5)	0.0193 (14)	
C15	0.4195 (2)	0.27110 (16)	0.7260 (6)	0.0252 (16)	
H15A	0.405490	0.273258	0.794794	0.038*	
H15B	0.431269	0.250936	0.719784	0.038*	
H15C	0.399037	0.274430	0.668949	0.038*	
C16	0.6196 (2)	0.34999 (17)	0.7895 (5)	0.0213 (14)	
C17	0.6670 (2)	0.35092 (17)	0.7983 (6)	0.0286 (16)	
H17A	0.678782	0.331942	0.773528	0.043*	
H17B	0.675002	0.354151	0.872503	0.043*	
H17C	0.677951	0.367257	0.754637	0.043*	
C18	0.5498 (2)	0.25712 (16)	0.9189 (5)	0.0208 (15)	
C19	0.5504 (3)	0.22453 (16)	0.9063 (5)	0.0306 (18)	
H19A	0.574786	0.218749	0.863130	0.046*	
H19B	0.524375	0.218023	0.871126	0.046*	
H19C	0.552416	0.215068	0.976207	0.046*	
N2	0.59476 (16)	0.31323 (12)	1.0972 (4)	0.0192 (12)	
C20	0.6248 (2)	0.30487 (17)	1.0282 (5)	0.0274 (17)	
H20	0.616422	0.295587	0.963831	0.033*	
C21	0.6667 (2)	0.3090 (2)	1.0460 (6)	0.039 (2)	
H21	0.686801	0.302801	0.994668	0.047*	
C22	0.6799 (2)	0.32238 (19)	1.1388 (6)	0.036 (2)	
H22	0.709048	0.325374	1.152790	0.043*	
C23	0.6495 (2)	0.33131 (17)	1.2112 (6)	0.0297 (17)	
H23	0.657353	0.340841	1.275592	0.036*	
C24	0.6079 (2)	0.32606 (16)	1.1878 (5)	0.0254 (16)	
H24	0.587203	0.331804	1.238401	0.030*	
C25	0.4366 (2)	0.31587 (16)	0.9917 (5)	0.0246 (16)	
H25	0.449451	0.329993	0.945790	0.030*	
C26	0.3935 (2)	0.31257 (17)	0.9890 (5)	0.0280 (17)	
H26	0.376923	0.324675	0.943172	0.034*	
C27	0.3742 (2)	0.29151 (17)	1.0533 (6)	0.0316 (18)	
H27	0.344487	0.288675	1.051419	0.038*	
C28	0.3991 (2)	0.27487 (17)	1.1198 (6)	0.0308 (18)	
H28	0.386806	0.260344	1.165143	0.037*	
C29	0.4425 (2)	0.27941 (16)	1.1204 (5)	0.0261 (16)	
H29	0.459466	0.267775	1.166747	0.031*	
C30	0.4637 (2)	0.34985 (15)	0.3626 (5)	0.0187 (14)	
H30	0.493365	0.351881	0.354525	0.022*	
C31	0.4386 (2)	0.34877 (15)	0.2726 (5)	0.0209 (14)	
H31	0.450918	0.350379	0.204127	0.025*	
C32	0.3959 (2)	0.34537 (16)	0.2832 (6)	0.0244 (15)	
H32	0.378207	0.344755	0.222390	0.029*	
C33	0.3787 (2)	0.34283 (16)	0.3842 (5)	0.0238 (16)	
H33	0.349239	0.340097	0.393566	0.029*	
C34	0.4053 (2)	0.34433 (15)	0.4702 (5)	0.0219 (15)	
H34	0.393338	0.342633	0.539074	0.026*	
F1	0.52458 (12)	0.29497 (9)	1.2121 (3)	0.0232 (9)	
N1	0.53052 (17)	0.35965 (12)	0.9354 (4)	0.0163 (12)	
N3	0.46126 (16)	0.29968 (12)	1.0577 (4)	0.0206 (12)	
N4	0.44722 (16)	0.34809 (12)	0.4618 (4)	0.0177 (12)	
O1	0.53120 (14)	0.32821 (10)	0.9290 (3)	0.0178 (10)	
O2	0.51946 (13)	0.35002 (10)	1.1099 (3)	0.0197 (10)	
O3	0.54669 (15)	0.41781 (11)	0.8852 (3)	0.0237 (11)	
O4	0.47337 (15)	0.39745 (11)	0.7996 (4)	0.0309 (12)	
O5	0.44128 (14)	0.37496 (11)	0.6630 (3)	0.0249 (11)	
O6	0.54797 (16)	0.40823 (11)	0.6633 (4)	0.0283 (12)	
O7	0.51501 (15)	0.38471 (11)	0.5322 (4)	0.0271 (11)	
O8	0.55440 (14)	0.29309 (10)	0.6355 (3)	0.0222 (11)	
O9	0.52547 (14)	0.32326 (11)	0.5146 (3)	0.0238 (11)	
O10	0.48483 (13)	0.29115 (10)	0.7812 (3)	0.0220 (10)	
O11	0.45184 (14)	0.31306 (11)	0.6447 (3)	0.0232 (11)	
O12	0.59983 (14)	0.37435 (10)	0.7909 (4)	0.0228 (11)	
O13	0.60280 (13)	0.32449 (10)	0.7846 (3)	0.0210 (10)	
O14	0.53657 (14)	0.26776 (10)	1.0068 (3)	0.0206 (10)	
O15	0.56241 (13)	0.27338 (10)	0.8435 (3)	0.0204 (10)	
O16	0.52158 (13)	0.34820 (10)	0.7222 (3)	0.0181 (10)	
N5	0.2858 (10)	0.2671 (6)	−0.144 (2)	0.155 (12)	0.5
C35	0.2940 (10)	0.2379 (6)	−0.163 (2)	0.127 (11)	0.5
H35	0.311010	0.232929	−0.222080	0.152*	0.5
C36	0.2797 (7)	0.2153 (5)	−0.1015 (17)	0.073 (6)	0.5
H36	0.283212	0.195132	−0.123399	0.088*	0.5
C37	0.2596 (7)	0.2218 (4)	−0.0060 (17)	0.063 (6)	0.5
H37	0.254457	0.206792	0.045608	0.076*	0.5
C38	0.2473 (17)	0.2515 (7)	0.0109 (14)	0.074 (6)	0.5
H38	0.227845	0.256511	0.065403	0.089*	0.5
C39	0.2643 (10)	0.2733 (5)	−0.054 (2)	0.095 (9)	0.5
H39	0.260803	0.293654	−0.034580	0.114*	0.5
O17	0.0851 (9)	0.0248 (6)	0.027 (3)	0.069 (12)	0.236 (17)
H17D	0.071299	0.026512	−0.031577	0.103*	0.236 (17)
H17E	0.105232	0.036950	0.018911	0.103*	0.236 (17)

### Hexa-µ-acetato-(µ-N,2-dioxodobenzene-1-carboximidato)fluorido-µ3-oxido-tripyridinetetrairon(III)–pyridine–water (1/1/0.24) (1-F) Atomic displacement parameters (Å^2^)

**Table:**

	*U*^11^	*U*^22^	*U*^33^	*U*^12^	*U*^13^	*U*^23^
Fe4	0.0181 (5)	0.0248 (5)	0.0081 (4)	−0.0004 (4)	−0.0020 (4)	0.0010 (4)
Fe3	0.0163 (5)	0.0266 (5)	0.0087 (4)	−0.0001 (4)	−0.0025 (4)	0.0009 (4)
Fe1	0.0181 (5)	0.0274 (5)	0.0077 (4)	0.0007 (4)	0.0004 (4)	0.0009 (4)
Fe2	0.0219 (5)	0.0256 (5)	0.0093 (5)	−0.0017 (4)	−0.0032 (4)	0.0008 (4)
C1	0.006 (3)	0.032 (4)	0.015 (3)	0.000 (3)	−0.001 (3)	−0.002 (3)
C2	0.009 (3)	0.028 (4)	0.017 (4)	0.005 (3)	−0.002 (2)	−0.002 (3)
C3	0.015 (4)	0.032 (4)	0.013 (3)	0.001 (3)	0.002 (3)	0.002 (3)
C4	0.020 (4)	0.036 (4)	0.020 (4)	0.001 (3)	0.000 (3)	−0.009 (3)
C5	0.035 (4)	0.030 (4)	0.025 (4)	0.002 (3)	0.001 (3)	−0.009 (3)
C6	0.033 (4)	0.025 (4)	0.021 (4)	−0.001 (3)	0.001 (3)	0.001 (3)
C7	0.016 (3)	0.033 (4)	0.012 (3)	0.002 (3)	−0.003 (3)	0.001 (3)
C8	0.031 (4)	0.043 (5)	0.011 (3)	0.006 (3)	−0.001 (3)	0.004 (3)
C9	0.036 (5)	0.098 (8)	0.025 (5)	0.029 (5)	−0.008 (4)	−0.013 (5)
C10	0.031 (4)	0.036 (4)	0.017 (4)	−0.009 (3)	−0.014 (3)	0.008 (3)
C11	0.060 (6)	0.080 (7)	0.026 (4)	−0.038 (5)	−0.010 (4)	0.023 (5)
C12	0.019 (3)	0.030 (4)	0.010 (3)	−0.002 (3)	0.003 (3)	−0.004 (3)
C13	0.034 (4)	0.035 (4)	0.006 (3)	0.008 (3)	0.006 (3)	0.001 (3)
C14	0.019 (3)	0.027 (4)	0.012 (3)	0.001 (3)	0.002 (3)	−0.005 (3)
C15	0.021 (4)	0.034 (4)	0.020 (4)	−0.004 (3)	−0.002 (3)	0.003 (3)
C16	0.023 (3)	0.036 (4)	0.005 (3)	−0.003 (3)	−0.002 (3)	−0.001 (3)
C17	0.019 (3)	0.046 (5)	0.021 (4)	−0.002 (3)	−0.001 (3)	−0.002 (3)
C18	0.018 (4)	0.037 (4)	0.008 (3)	−0.004 (3)	0.002 (3)	0.000 (3)
C19	0.052 (5)	0.026 (4)	0.013 (4)	−0.006 (4)	0.010 (3)	0.001 (3)
N2	0.019 (3)	0.030 (3)	0.009 (3)	−0.003 (2)	−0.001 (2)	0.001 (2)
C20	0.025 (4)	0.050 (5)	0.007 (3)	−0.002 (3)	−0.003 (3)	−0.004 (3)
C21	0.023 (4)	0.077 (6)	0.018 (4)	−0.002 (4)	0.005 (3)	−0.007 (4)
C22	0.017 (4)	0.062 (6)	0.029 (4)	−0.009 (4)	−0.007 (3)	0.003 (4)
C23	0.029 (4)	0.039 (5)	0.021 (4)	−0.006 (3)	−0.008 (3)	0.003 (3)
C24	0.028 (4)	0.034 (4)	0.015 (4)	−0.001 (3)	−0.003 (3)	−0.001 (3)
C25	0.024 (4)	0.032 (4)	0.018 (4)	0.002 (3)	−0.001 (3)	−0.005 (3)
C26	0.025 (4)	0.040 (5)	0.019 (4)	0.013 (3)	−0.004 (3)	−0.008 (3)
C27	0.024 (4)	0.043 (5)	0.028 (4)	−0.003 (3)	−0.001 (3)	−0.016 (4)
C28	0.025 (4)	0.040 (5)	0.027 (4)	−0.005 (3)	0.006 (3)	−0.007 (4)
C29	0.031 (4)	0.033 (4)	0.014 (3)	−0.001 (3)	0.004 (3)	−0.004 (3)
C30	0.014 (3)	0.025 (4)	0.017 (3)	0.000 (3)	−0.002 (3)	0.006 (3)
C31	0.023 (3)	0.028 (4)	0.012 (3)	0.001 (3)	−0.001 (3)	−0.002 (3)
C32	0.024 (4)	0.034 (4)	0.015 (3)	0.003 (3)	−0.005 (3)	−0.002 (3)
C33	0.017 (4)	0.042 (5)	0.012 (3)	0.000 (3)	0.000 (3)	0.001 (3)
C34	0.019 (3)	0.036 (4)	0.010 (3)	−0.001 (3)	0.004 (3)	−0.001 (3)
F1	0.024 (2)	0.038 (2)	0.0072 (18)	0.0010 (17)	0.0008 (15)	0.0027 (17)
N1	0.016 (3)	0.021 (3)	0.012 (3)	0.000 (2)	−0.002 (2)	0.000 (2)
N3	0.023 (3)	0.025 (3)	0.014 (3)	−0.001 (2)	0.001 (2)	−0.004 (2)
N4	0.015 (3)	0.028 (3)	0.010 (3)	0.003 (2)	−0.001 (2)	0.004 (2)
O1	0.020 (2)	0.021 (3)	0.012 (2)	−0.001 (2)	−0.0031 (17)	−0.004 (2)
O2	0.019 (2)	0.031 (3)	0.009 (2)	−0.001 (2)	0.0002 (18)	0.001 (2)
O3	0.033 (3)	0.026 (3)	0.012 (2)	−0.004 (2)	−0.001 (2)	0.001 (2)
O4	0.029 (3)	0.039 (3)	0.025 (3)	0.006 (2)	−0.010 (2)	−0.008 (2)
O5	0.024 (3)	0.043 (3)	0.008 (2)	0.010 (2)	−0.0059 (19)	−0.004 (2)
O6	0.035 (3)	0.032 (3)	0.018 (3)	−0.009 (2)	−0.004 (2)	0.005 (2)
O7	0.031 (3)	0.035 (3)	0.015 (2)	−0.008 (2)	−0.009 (2)	0.004 (2)
O8	0.028 (3)	0.029 (3)	0.010 (2)	0.002 (2)	−0.001 (2)	0.001 (2)
O9	0.022 (2)	0.037 (3)	0.012 (2)	0.009 (2)	−0.0026 (19)	0.004 (2)
O10	0.023 (2)	0.030 (3)	0.014 (2)	−0.004 (2)	−0.003 (2)	0.004 (2)
O11	0.027 (3)	0.031 (3)	0.012 (2)	−0.002 (2)	−0.006 (2)	0.005 (2)
O12	0.026 (3)	0.026 (3)	0.016 (2)	−0.001 (2)	0.000 (2)	0.000 (2)
O13	0.022 (2)	0.028 (3)	0.014 (2)	−0.003 (2)	0.0019 (19)	0.000 (2)
O14	0.024 (2)	0.028 (3)	0.010 (2)	0.004 (2)	0.0004 (18)	−0.0002 (19)
O15	0.019 (2)	0.029 (3)	0.012 (2)	−0.001 (2)	−0.0008 (19)	0.001 (2)
O16	0.015 (2)	0.030 (3)	0.009 (2)	−0.002 (2)	0.0010 (18)	−0.001 (2)
N5	0.184 (16)	0.140 (16)	0.141 (16)	−0.005 (12)	0.035 (13)	−0.009 (12)
C35	0.158 (16)	0.101 (15)	0.121 (16)	−0.005 (12)	0.011 (13)	−0.011 (12)
C36	0.089 (12)	0.067 (11)	0.064 (11)	−0.019 (10)	0.023 (10)	−0.008 (9)
C37	0.066 (11)	0.042 (10)	0.080 (12)	−0.014 (9)	0.002 (10)	−0.005 (9)
C38	0.077 (12)	0.067 (10)	0.077 (10)	−0.012 (9)	0.003 (14)	0.005 (14)
C39	0.112 (15)	0.073 (13)	0.099 (14)	−0.026 (11)	0.017 (12)	0.000 (11)
O17	0.063 (18)	0.047 (17)	0.09 (2)	−0.016 (13)	0.008 (15)	−0.009 (15)

### Hexa-µ-acetato-(µ-N,2-dioxodobenzene-1-carboximidato)fluorido-µ3-oxido-tripyridinetetrairon(III)–pyridine–water (1/1/0.24) (1-F) Geometric parameters (Å, º)

**Table:**

Fe4—O16	1.890 (5)	C15—H15C	0.9800
Fe4—O15	2.016 (5)	C16—O12	1.258 (8)
Fe4—O13	2.021 (4)	C16—O13	1.262 (8)
Fe4—O10	2.023 (4)	C16—C17	1.505 (9)
Fe4—O8	2.034 (4)	C17—H17A	0.9800
Fe4—O1	2.056 (4)	C17—H17B	0.9800
Fe3—O16	1.872 (4)	C17—H17C	0.9800
Fe3—O9	1.997 (5)	C18—O15	1.263 (8)
Fe3—O5	2.020 (5)	C18—O14	1.276 (8)
Fe3—O11	2.024 (5)	C18—C19	1.469 (10)
Fe3—O7	2.037 (5)	C19—H19A	0.9800
Fe3—N4	2.170 (5)	C19—H19B	0.9800
Fe1—F1	1.843 (4)	C19—H19C	0.9800
Fe1—O2	1.978 (5)	N2—C20	1.343 (8)
Fe1—O14	1.987 (4)	N2—C24	1.345 (8)
Fe1—O1	2.043 (5)	C20—C21	1.357 (9)
Fe1—N3	2.133 (5)	C20—H20	0.9500
Fe1—N2	2.171 (5)	C21—C22	1.379 (10)
Fe2—O3	1.902 (5)	C21—H21	0.9500
Fe2—O16	1.925 (4)	C22—C23	1.384 (10)
Fe2—O6	2.021 (5)	C22—H22	0.9500
Fe2—O12	2.057 (5)	C23—C24	1.372 (10)
Fe2—N1	2.083 (5)	C23—H23	0.9500
Fe2—O4	2.084 (5)	C24—H24	0.9500
C1—O2	1.287 (8)	C25—N3	1.352 (9)
C1—N1	1.329 (8)	C25—C26	1.374 (9)
C1—C2	1.461 (9)	C25—H25	0.9500
C2—C7	1.412 (9)	C26—C27	1.385 (11)
C2—C3	1.412 (9)	C26—H26	0.9500
C3—C4	1.369 (10)	C27—C28	1.372 (11)
C3—H3	0.9500	C27—H27	0.9500
C4—C5	1.389 (10)	C28—C29	1.389 (10)
C4—H4	0.9500	C28—H28	0.9500
C5—C6	1.382 (10)	C29—N3	1.343 (9)
C5—H5	0.9500	C29—H29	0.9500
C6—C7	1.403 (10)	C30—N4	1.357 (8)
C6—H6	0.9500	C30—C31	1.386 (9)
C7—O3	1.325 (8)	C30—H30	0.9500
C8—O4	1.252 (8)	C31—C32	1.367 (9)
C8—O5	1.274 (9)	C31—H31	0.9500
C8—C9	1.501 (11)	C32—C33	1.388 (9)
C9—H9A	0.9800	C32—H32	0.9500
C9—H9B	0.9800	C33—C34	1.373 (9)
C9—H9C	0.9800	C33—H33	0.9500
C10—O7	1.243 (8)	C34—N4	1.344 (8)
C10—O6	1.262 (8)	C34—H34	0.9500
C10—C11	1.499 (10)	N1—O1	1.411 (6)
C11—H11A	0.9800	N5—C35	1.35 (2)
C11—H11B	0.9800	N5—C39	1.35 (2)
C11—H11C	0.9800	C35—C36	1.35 (2)
C12—O9	1.253 (8)	C35—H35	0.9500
C12—O8	1.261 (8)	C36—C37	1.39 (2)
C12—C13	1.504 (9)	C36—H36	0.9500
C13—H13A	0.9800	C37—C38	1.40 (3)
C13—H13B	0.9800	C37—H37	0.9500
C13—H13C	0.9800	C38—C39	1.39 (3)
C14—O10	1.259 (8)	C38—H38	0.9500
C14—O11	1.267 (8)	C39—H39	0.9500
C14—C15	1.502 (9)	O17—H17D	0.8564
C15—H15A	0.9800	O17—H17E	0.8446
C15—H15B	0.9800		
			
O16—Fe4—O15	178.35 (19)	H15A—C15—H15C	109.5
O16—Fe4—O13	95.35 (18)	H15B—C15—H15C	109.5
O15—Fe4—O13	84.96 (18)	O12—C16—O13	125.2 (6)
O16—Fe4—O10	95.84 (18)	O12—C16—C17	118.1 (6)
O15—Fe4—O10	83.89 (18)	O13—C16—C17	116.7 (6)
O13—Fe4—O10	168.72 (19)	C16—C17—H17A	109.5
O16—Fe4—O8	94.97 (18)	C16—C17—H17B	109.5
O15—Fe4—O8	86.66 (18)	H17A—C17—H17B	109.5
O13—Fe4—O8	87.52 (18)	C16—C17—H17C	109.5
O10—Fe4—O8	90.07 (18)	H17A—C17—H17C	109.5
O16—Fe4—O1	89.18 (18)	H17B—C17—H17C	109.5
O15—Fe4—O1	89.19 (18)	O15—C18—O14	122.8 (6)
O13—Fe4—O1	91.19 (18)	O15—C18—C19	119.2 (6)
O10—Fe4—O1	90.42 (18)	O14—C18—C19	118.0 (6)
O8—Fe4—O1	175.74 (18)	C18—C19—H19A	109.5
O16—Fe3—O9	94.78 (18)	C18—C19—H19B	109.5
O16—Fe3—O5	96.68 (18)	H19A—C19—H19B	109.5
O9—Fe3—O5	168.52 (18)	C18—C19—H19C	109.5
O16—Fe3—O11	95.43 (18)	H19A—C19—H19C	109.5
O9—Fe3—O11	91.8 (2)	H19B—C19—H19C	109.5
O5—Fe3—O11	87.8 (2)	C20—N2—C24	116.7 (6)
O16—Fe3—O7	96.08 (19)	C20—N2—Fe1	125.0 (4)
O9—Fe3—O7	87.2 (2)	C24—N2—Fe1	118.3 (5)
O5—Fe3—O7	90.9 (2)	N2—C20—C21	123.2 (6)
O11—Fe3—O7	168.49 (18)	N2—C20—H20	118.4
O16—Fe3—N4	178.5 (2)	C21—C20—H20	118.4
O9—Fe3—N4	84.23 (19)	C20—C21—C22	119.7 (7)
O5—Fe3—N4	84.33 (18)	C20—C21—H21	120.1
O11—Fe3—N4	83.47 (19)	C22—C21—H21	120.1
O7—Fe3—N4	85.02 (19)	C21—C22—C23	118.3 (7)
F1—Fe1—O2	93.63 (18)	C21—C22—H22	120.9
F1—Fe1—O14	97.73 (18)	C23—C22—H22	120.9
O2—Fe1—O14	168.62 (18)	C24—C23—C22	118.5 (7)
F1—Fe1—O1	170.62 (18)	C24—C23—H23	120.8
O2—Fe1—O1	77.18 (18)	C22—C23—H23	120.8
O14—Fe1—O1	91.44 (17)	N2—C24—C23	123.6 (7)
F1—Fe1—N3	89.35 (19)	N2—C24—H24	118.2
O2—Fe1—N3	93.10 (19)	C23—C24—H24	118.2
O14—Fe1—N3	87.6 (2)	N3—C25—C26	122.2 (7)
O1—Fe1—N3	93.0 (2)	N3—C25—H25	118.9
F1—Fe1—N2	87.15 (18)	C26—C25—H25	118.9
O2—Fe1—N2	88.72 (19)	C25—C26—C27	119.8 (7)
O14—Fe1—N2	91.2 (2)	C25—C26—H26	120.1
O1—Fe1—N2	90.74 (19)	C27—C26—H26	120.1
N3—Fe1—N2	176.2 (2)	C28—C27—C26	118.3 (7)
O3—Fe2—O16	171.98 (19)	C28—C27—H27	120.8
O3—Fe2—O6	92.3 (2)	C26—C27—H27	120.8
O16—Fe2—O6	95.12 (19)	C27—C28—C29	119.5 (7)
O3—Fe2—O12	91.36 (19)	C27—C28—H28	120.2
O16—Fe2—O12	92.38 (18)	C29—C28—H28	120.2
O6—Fe2—O12	85.01 (19)	N3—C29—C28	122.3 (7)
O3—Fe2—N1	86.4 (2)	N3—C29—H29	118.9
O16—Fe2—N1	86.6 (2)	C28—C29—H29	118.9
O6—Fe2—N1	174.8 (2)	N4—C30—C31	122.1 (6)
O12—Fe2—N1	90.0 (2)	N4—C30—H30	118.9
O3—Fe2—O4	85.9 (2)	C31—C30—H30	118.9
O16—Fe2—O4	90.72 (19)	C32—C31—C30	119.4 (6)
O6—Fe2—O4	92.3 (2)	C32—C31—H31	120.3
O12—Fe2—O4	176.1 (2)	C30—C31—H31	120.3
N1—Fe2—O4	92.6 (2)	C31—C32—C33	119.1 (6)
O2—C1—N1	120.3 (6)	C31—C32—H32	120.5
O2—C1—C2	119.7 (6)	C33—C32—H32	120.5
N1—C1—C2	120.0 (6)	C34—C33—C32	118.7 (6)
C7—C2—C3	118.3 (6)	C34—C33—H33	120.6
C7—C2—C1	124.5 (6)	C32—C33—H33	120.6
C3—C2—C1	116.9 (6)	N4—C34—C33	123.3 (6)
C4—C3—C2	122.0 (6)	N4—C34—H34	118.4
C4—C3—H3	119.0	C33—C34—H34	118.4
C2—C3—H3	119.0	C1—N1—O1	111.7 (5)
C3—C4—C5	119.5 (6)	C1—N1—Fe2	129.6 (4)
C3—C4—H4	120.2	O1—N1—Fe2	118.7 (3)
C5—C4—H4	120.2	C29—N3—C25	117.9 (6)
C6—C5—C4	120.2 (7)	C29—N3—Fe1	119.5 (5)
C6—C5—H5	119.9	C25—N3—Fe1	122.4 (5)
C4—C5—H5	119.9	C34—N4—C30	117.4 (5)
C5—C6—C7	121.2 (7)	C34—N4—Fe3	120.9 (4)
C5—C6—H6	119.4	C30—N4—Fe3	121.7 (4)
C7—C6—H6	119.4	N1—O1—Fe1	113.7 (3)
O3—C7—C6	118.0 (6)	N1—O1—Fe4	114.3 (3)
O3—C7—C2	123.1 (6)	Fe1—O1—Fe4	131.7 (2)
C6—C7—C2	118.8 (6)	C1—O2—Fe1	116.5 (4)
O4—C8—O5	124.8 (7)	C7—O3—Fe2	132.4 (4)
O4—C8—C9	119.4 (7)	C8—O4—Fe2	134.9 (5)
O5—C8—C9	115.9 (7)	C8—O5—Fe3	130.5 (4)
C8—C9—H9A	109.5	C10—O6—Fe2	132.7 (5)
C8—C9—H9B	109.5	C10—O7—Fe3	131.9 (4)
H9A—C9—H9B	109.5	C12—O8—Fe4	132.9 (4)
C8—C9—H9C	109.5	C12—O9—Fe3	129.9 (4)
H9A—C9—H9C	109.5	C14—O10—Fe4	129.0 (4)
H9B—C9—H9C	109.5	C14—O11—Fe3	134.5 (4)
O7—C10—O6	124.9 (7)	C16—O12—Fe2	132.4 (4)
O7—C10—C11	117.7 (6)	C16—O13—Fe4	131.3 (4)
O6—C10—C11	117.3 (6)	C18—O14—Fe1	138.3 (5)
C10—C11—H11A	109.5	C18—O15—Fe4	133.9 (4)
C10—C11—H11B	109.5	Fe3—O16—Fe4	120.9 (2)
H11A—C11—H11B	109.5	Fe3—O16—Fe2	121.7 (2)
C10—C11—H11C	109.5	Fe4—O16—Fe2	117.4 (2)
H11A—C11—H11C	109.5	C35—N5—C39	116 (2)
H11B—C11—H11C	109.5	C36—C35—N5	124 (2)
O9—C12—O8	125.4 (6)	C36—C35—H35	117.9
O9—C12—C13	117.8 (6)	N5—C35—H35	117.9
O8—C12—C13	116.8 (6)	C35—C36—C37	119 (2)
C12—C13—H13A	109.5	C35—C36—H36	120.4
C12—C13—H13B	109.5	C37—C36—H36	120.4
H13A—C13—H13B	109.5	C36—C37—C38	117.3 (19)
C12—C13—H13C	109.5	C36—C37—H37	121.4
H13A—C13—H13C	109.5	C38—C37—H37	121.4
H13B—C13—H13C	109.5	C39—C38—C37	118 (3)
O10—C14—O11	125.0 (6)	C39—C38—H38	120.9
O10—C14—C15	117.2 (6)	C37—C38—H38	120.9
O11—C14—C15	117.7 (6)	N5—C39—C38	123 (2)
C14—C15—H15A	109.5	N5—C39—H39	118.5
C14—C15—H15B	109.5	C38—C39—H39	118.5
H15A—C15—H15B	109.5	H17D—O17—H17E	103.3
C14—C15—H15C	109.5		

### Hexa-µ-acetato-(µ-N,2-dioxodobenzene-1-carboximidato)fluorido-µ3-oxido-tripyridinetetrairon(III)–pyridine–water (1/1/0.24) (1-F) Hydrogen-bond geometry (Å, º)

**Table:**

*D*—H···*A*	*D*—H	H···*A*	*D*···*A*	*D*—H···*A*
C11—H11*A*···N5^i^	0.98	2.37	3.23 (3)	146
C13—H13*A*···F1^ii^	0.98	2.54	3.389 (7)	145
C15—H15*B*···F1^iii^	0.98	2.49	3.454 (8)	168
C17—H17*A*···O17^iv^	0.98	2.60	3.42 (3)	142
C19—H19*B*···F1^iii^	0.98	2.60	3.520 (9)	156
C19—H19*C*···O11^v^	0.98	2.47	3.446 (8)	172
C20—H20···O13	0.95	2.64	3.269 (8)	124
C20—H20···O15	0.95	2.49	3.365 (8)	153
C23—H23···O5^i^	0.95	2.55	3.473 (9)	164
C25—H25···O1	0.95	2.60	3.149 (8)	117
C26—H26···O6^vi^	0.95	2.64	3.431 (8)	141
C29—H29···F1	0.95	2.46	2.930 (8)	110
C31—H31···O2^ii^	0.95	2.48	3.282 (8)	143
C39—H39···O6^vii^	0.95	2.54	3.27 (2)	134
O17—H17*E*···O13^viii^	0.84	2.15	2.97 (3)	164

Symmetry codes: (i) *x*+1/4, −*y*+3/4, *z*+3/4; (ii) *x*, *y*, *z*−1; (iii) −*x*+1, −*y*+1/2, *z*−1/2; (iv) −*x*+3/4, *y*+1/4, *z*+3/4; (v) −*x*+1, −*y*+1/2, *z*+1/2; (vi) *x*−1/4, −*y*+3/4, *z*+1/4; (vii) *x*−1/4, −*y*+3/4, *z*−3/4; (viii) −*x*+3/4, *y*−1/4, *z*−3/4.

### Hexa-µ-acetato-chlorido(µ-N,2-dioxodobenzene-1-carboximidato)-µ3-oxido-tetrairon(III)–water (1/1) (1-Cl) Crystal data

**Table:**

[Fe_4_(C_2_H_3_O_2_)_6_(C_7_H_4_O_3_)ClO(C_5_H_5_N)_3_]·H_2_O	*D*_x_ = 1.639 Mg m^−^^3^
*M**_r_* = 1034.54	Melting point: 238 K
Monoclinic, *P*2_1_	Mo *K*α radiation, λ = 0.71073 Å
*a* = 11.8460 (6) Å	Cell parameters from 9996 reflections
*b* = 15.5041 (7) Å	θ = 2.3–24.9°
*c* = 12.6425 (6) Å	µ = 1.50 mm^−^^1^
β = 115.449 (1)°	*T* = 100 K
*V* = 2096.64 (17) Å^3^	Block, dark brown
*Z* = 2	0.28 × 0.28 × 0.14 mm
*F*(000) = 1056	

### Hexa-µ-acetato-chlorido(µ-N,2-dioxodobenzene-1-carboximidato)-µ3-oxido-tetrairon(III)–water (1/1) (1-Cl) Data collection

**Table:**

Bruker APEXII CCD diffractometer	7877 reflections with *I* > 2σ(*I*)
φ and ω scans	*R*_int_ = 0.048
Absorption correction: multi-scan (SADABS; Krause *et al.*, 2015)	θ_max_ = 26.5°, θ_min_ = 1.9°
*T*_min_ = 0.688, *T*_max_ = 0.745	*h* = −14→14
52843 measured reflections	*k* = −19→19
8621 independent reflections	*l* = −15→15

### Hexa-µ-acetato-chlorido(µ-N,2-dioxodobenzene-1-carboximidato)-µ3-oxido-tetrairon(III)–water (1/1) (1-Cl) Refinement

**Table:**

Refinement on *F*^2^	Secondary atom site location: difference Fourier map
Least-squares matrix: full	Hydrogen site location: mixed
*R*[*F*^2^ > 2σ(*F*^2^)] = 0.033	H atoms treated by a mixture of independent and constrained refinement
*wR*(*F*^2^) = 0.071	*w* = 1/[σ^2^(*F*_o_^2^) + (0.0296*P*)^2^ + 1.4211*P*] where *P* = (*F*_o_^2^ + 2*F*_c_^2^)/3
*S* = 1.06	(Δ/σ)_max_ < 0.001
8621 reflections	Δρ_max_ = 0.43 e Å^−^^3^
570 parameters	Δρ_min_ = −0.34 e Å^−^^3^
7 restraints	Absolute structure: Refined as an inversion twin
Primary atom site location: dual	Absolute structure parameter: 0.014 (16)

### Hexa-µ-acetato-chlorido(µ-N,2-dioxodobenzene-1-carboximidato)-µ3-oxido-tetrairon(III)–water (1/1) (1-Cl) Special details

**Table:**

Geometry. All esds (except the esd in the dihedral angle between two l.s. planes) are estimated using the full covariance matrix. The cell esds are taken into account individually in the estimation of esds in distances, angles and torsion angles; correlations between esds in cell parameters are only used when they are defined by crystal symmetry. An approximate (isotropic) treatment of cell esds is used for estimating esds involving l.s. planes.
Refinement. Refined as a two-component inversion twin. A lattice water containing O17 was refined over two sites with a PART command and the occupancies were refined using a free variable. H atoms were found on the difference map and refined with DFIX and DANG commands. Minor disorder of the pyridine containing N4 was not refined and is likely due to the disorder of the lattice water.

### Hexa-µ-acetato-chlorido(µ-N,2-dioxodobenzene-1-carboximidato)-µ3-oxido-tetrairon(III)–water (1/1) (1-Cl) Fractional atomic coordinates and isotropic or equivalent isotropic displacement parameters (Å^2^)

**Table:**

	*x*	*y*	*z*	*U*_iso_*/*U*_eq_	Occ. (<1)
Cl1	−0.46846 (12)	0.72584 (9)	0.49062 (11)	0.0297 (3)	
Fe2	0.12283 (6)	0.75827 (4)	0.96279 (6)	0.01283 (15)	
Fe4	0.08443 (6)	0.69044 (4)	0.70471 (6)	0.01401 (15)	
Fe1	−0.25727 (6)	0.71218 (4)	0.60363 (6)	0.01778 (16)	
Fe3	0.28619 (6)	0.84403 (4)	0.83141 (6)	0.01368 (15)	
C1	−0.1641 (4)	0.7441 (3)	0.8433 (4)	0.0134 (10)	
C2	−0.1680 (4)	0.7585 (3)	0.9578 (4)	0.0132 (9)	
C3	−0.2869 (5)	0.7669 (3)	0.9550 (5)	0.0205 (11)	
H3	−0.358389	0.767725	0.881605	0.025*	
C4	−0.3023 (5)	0.7740 (4)	1.0561 (5)	0.0254 (12)	
H4	−0.383326	0.780059	1.053180	0.031*	
C5	−0.1969 (5)	0.7722 (3)	1.1630 (5)	0.0245 (12)	
H5	−0.206497	0.775901	1.233706	0.029*	
C6	−0.0793 (5)	0.7651 (3)	1.1678 (4)	0.0195 (11)	
H6	−0.008649	0.764313	1.241787	0.023*	
C7	−0.0619 (5)	0.7589 (3)	1.0642 (4)	0.0155 (9)	
C16	0.1488 (5)	0.5709 (3)	0.9094 (4)	0.0188 (11)	
C17	0.1697 (6)	0.4792 (4)	0.9525 (5)	0.0346 (14)	
H17A	0.187704	0.477933	1.035774	0.052*	
H17B	0.240496	0.454736	0.942055	0.052*	
H17C	0.094403	0.445083	0.907825	0.052*	
C10	0.3908 (5)	0.8117 (3)	1.0937 (4)	0.0198 (11)	
C11	0.5042 (5)	0.8197 (4)	1.2090 (5)	0.0342 (15)	
H11A	0.575040	0.840336	1.195386	0.051*	
H11B	0.524424	0.763221	1.247435	0.051*	
H11C	0.486972	0.860736	1.259151	0.051*	
C8	0.1256 (5)	0.9514 (3)	0.9086 (4)	0.0171 (10)	
C9	0.0811 (6)	1.0401 (3)	0.9203 (5)	0.0294 (13)	
H9A	−0.005582	1.037004	0.910108	0.044*	
H9B	0.085787	1.078003	0.860182	0.044*	
H9C	0.134156	1.063301	0.998078	0.044*	
C14	0.1095 (4)	0.8432 (3)	0.5730 (4)	0.0168 (10)	
C15	0.0728 (5)	0.8871 (4)	0.4569 (4)	0.0276 (13)	
H15A	−0.001668	0.859109	0.397687	0.041*	
H15B	0.141664	0.882858	0.433746	0.041*	
H15C	0.054627	0.947974	0.463693	0.041*	
C12	0.3554 (5)	0.6848 (3)	0.7421 (4)	0.0198 (10)	
C13	0.4548 (5)	0.6284 (4)	0.7343 (5)	0.0305 (13)	
H13A	0.502815	0.661681	0.701670	0.046*	
H13B	0.415516	0.578984	0.683336	0.046*	
H13C	0.510937	0.607745	0.812649	0.046*	
C18	−0.1213 (5)	0.6292 (3)	0.4774 (4)	0.0192 (11)	
C19	−0.1451 (6)	0.5866 (4)	0.3639 (4)	0.0327 (14)	
H19A	−0.202946	0.538237	0.350530	0.049*	
H19B	−0.066066	0.565132	0.366613	0.049*	
H19C	−0.181915	0.628372	0.299934	0.049*	
C30	0.4475 (5)	1.0113 (4)	0.8995 (5)	0.0286 (13)	
H30	0.412411	1.015750	0.954165	0.034*	
C31	0.5223 (5)	1.0776 (4)	0.8928 (5)	0.0348 (14)	
H31	0.537259	1.126859	0.941708	0.042*	
C32	0.5751 (6)	1.0719 (4)	0.8145 (5)	0.0385 (16)	
H32	0.625285	1.117159	0.807069	0.046*	
C33	0.5522 (5)	0.9987 (4)	0.7481 (5)	0.0323 (14)	
H33	0.589537	0.991713	0.695426	0.039*	
C34	0.4756 (5)	0.9350 (4)	0.7568 (4)	0.0247 (12)	
H34	0.459736	0.885358	0.708364	0.030*	
C20	−0.1920 (5)	0.5370 (3)	0.7353 (4)	0.0241 (12)	
H20	−0.125234	0.570544	0.789816	0.029*	
C21	−0.2018 (5)	0.4517 (4)	0.7622 (5)	0.0286 (13)	
H21	−0.143450	0.427567	0.833943	0.034*	
C22	−0.2970 (5)	0.4025 (4)	0.6837 (5)	0.0291 (13)	
H22	−0.305480	0.343694	0.699843	0.035*	
C23	−0.3809 (6)	0.4403 (4)	0.5798 (5)	0.0368 (15)	
H23	−0.447467	0.407585	0.523709	0.044*	
C24	−0.3659 (5)	0.5263 (4)	0.5595 (5)	0.0302 (13)	
H24	−0.423884	0.552129	0.488893	0.036*	
C25	−0.2080 (8)	0.9070 (4)	0.6453 (6)	0.051 (2)	
H25	−0.203735	0.891707	0.719715	0.062*	
C26	−0.1904 (9)	0.9928 (5)	0.6251 (7)	0.073 (3)	
H26	−0.175730	1.035107	0.683979	0.088*	
C27	−0.1947 (8)	1.0154 (6)	0.5189 (8)	0.067 (3)	
H27	−0.181755	1.073511	0.502907	0.080*	
C28	−0.2185 (6)	0.9516 (6)	0.4351 (7)	0.052 (2)	
H28	−0.223206	0.965351	0.360092	0.063*	
C29	−0.2350 (6)	0.8685 (4)	0.4624 (5)	0.0359 (15)	
H29	−0.250573	0.825214	0.404569	0.043*	
N1	−0.0574 (4)	0.7339 (2)	0.8375 (3)	0.0140 (8)	
N4	0.4229 (4)	0.9407 (3)	0.8313 (4)	0.0196 (9)	
N2	−0.2723 (4)	0.5746 (3)	0.6361 (4)	0.0206 (9)	
N3	−0.2304 (4)	0.8448 (3)	0.5661 (4)	0.0254 (10)	
O1	−0.0757 (3)	0.7135 (2)	0.7220 (2)	0.0147 (7)	
O2	−0.2688 (3)	0.7401 (2)	0.7496 (3)	0.0182 (8)	
O3	0.0537 (3)	0.7516 (2)	1.0747 (3)	0.0164 (7)	
O16	0.1694 (3)	0.7641 (2)	0.8334 (3)	0.0143 (7)	
O12	0.1617 (3)	0.6289 (2)	0.9829 (3)	0.0205 (8)	
O13	0.1159 (3)	0.5825 (2)	0.8016 (3)	0.0208 (8)	
O6	0.2944 (3)	0.7771 (2)	1.0935 (3)	0.0216 (8)	
O7	0.4011 (3)	0.8406 (2)	1.0054 (3)	0.0204 (7)	
O4	0.0908 (3)	0.8899 (2)	0.9526 (3)	0.0191 (8)	
O5	0.1963 (3)	0.9458 (2)	0.8581 (3)	0.0196 (7)	
O10	0.0420 (3)	0.7825 (2)	0.5788 (3)	0.0178 (7)	
O11	0.2077 (3)	0.8700 (2)	0.6584 (3)	0.0182 (8)	
O8	0.2429 (3)	0.6626 (2)	0.6858 (3)	0.0207 (8)	
O9	0.3926 (3)	0.7530 (2)	0.8034 (3)	0.0192 (7)	
O15	−0.0180 (3)	0.6149 (2)	0.5639 (3)	0.0213 (8)	
O14	−0.2075 (3)	0.6755 (2)	0.4795 (3)	0.0232 (8)	
O17	0.2126 (7)	0.6698 (6)	0.2870 (8)	0.076 (4)	0.715 (13)
H17D	0.289 (5)	0.680 (9)	0.332 (10)	0.115*	0.715 (13)
H17E	0.181 (10)	0.704 (7)	0.230 (8)	0.115*	0.715 (13)
O17A	0.2102 (17)	0.6965 (16)	0.3790 (16)	0.061 (7)	0.285 (13)
H17F	0.269 (19)	0.670 (18)	0.434 (16)	0.091*	0.285 (13)
H17G	0.16 (2)	0.71 (2)	0.41 (2)	0.091*	0.285 (13)

### Hexa-µ-acetato-chlorido(µ-N,2-dioxodobenzene-1-carboximidato)-µ3-oxido-tetrairon(III)–water (1/1) (1-Cl) Atomic displacement parameters (Å^2^)

**Table:**

	*U*^11^	*U*^22^	*U*^33^	*U*^12^	*U*^13^	*U*^23^
Cl1	0.0217 (6)	0.0401 (8)	0.0221 (6)	−0.0020 (6)	0.0044 (5)	0.0074 (6)
Fe2	0.0166 (3)	0.0122 (3)	0.0120 (3)	−0.0007 (3)	0.0084 (3)	−0.0021 (3)
Fe4	0.0166 (3)	0.0132 (3)	0.0144 (3)	−0.0023 (3)	0.0087 (3)	−0.0046 (3)
Fe1	0.0165 (3)	0.0243 (4)	0.0122 (3)	−0.0019 (3)	0.0058 (3)	0.0018 (3)
Fe3	0.0163 (3)	0.0135 (3)	0.0128 (3)	−0.0024 (3)	0.0078 (3)	−0.0021 (3)
C1	0.017 (2)	0.010 (2)	0.016 (2)	0.0008 (19)	0.009 (2)	0.0005 (17)
C2	0.021 (2)	0.008 (2)	0.015 (2)	0.000 (2)	0.012 (2)	0.0008 (19)
C3	0.021 (3)	0.017 (3)	0.028 (3)	−0.004 (2)	0.015 (2)	−0.005 (2)
C4	0.024 (3)	0.029 (3)	0.033 (3)	−0.006 (2)	0.021 (3)	−0.011 (2)
C5	0.034 (3)	0.024 (3)	0.024 (3)	−0.002 (2)	0.020 (3)	−0.004 (2)
C6	0.031 (3)	0.017 (3)	0.015 (2)	−0.005 (2)	0.015 (2)	−0.001 (2)
C7	0.026 (3)	0.006 (2)	0.019 (2)	0.001 (2)	0.014 (2)	−0.0007 (19)
C16	0.017 (3)	0.015 (3)	0.024 (3)	0.000 (2)	0.009 (2)	−0.002 (2)
C17	0.054 (4)	0.018 (3)	0.028 (3)	0.005 (3)	0.013 (3)	0.002 (2)
C10	0.020 (3)	0.023 (3)	0.021 (3)	0.007 (2)	0.012 (2)	0.000 (2)
C11	0.022 (3)	0.066 (5)	0.017 (3)	−0.001 (3)	0.010 (2)	−0.002 (3)
C8	0.021 (3)	0.015 (2)	0.014 (2)	0.001 (2)	0.006 (2)	−0.0025 (19)
C9	0.042 (3)	0.015 (3)	0.038 (3)	0.006 (3)	0.024 (3)	0.001 (2)
C14	0.021 (3)	0.019 (2)	0.017 (2)	−0.002 (2)	0.014 (2)	−0.002 (2)
C15	0.031 (3)	0.037 (3)	0.012 (3)	−0.012 (3)	0.007 (2)	0.001 (2)
C12	0.021 (3)	0.022 (3)	0.020 (2)	−0.003 (2)	0.012 (2)	−0.004 (2)
C13	0.028 (3)	0.025 (3)	0.045 (4)	−0.001 (2)	0.022 (3)	−0.010 (3)
C18	0.027 (3)	0.015 (3)	0.016 (3)	−0.009 (2)	0.009 (2)	−0.001 (2)
C19	0.052 (4)	0.026 (3)	0.015 (3)	0.006 (3)	0.009 (3)	−0.005 (2)
C30	0.029 (3)	0.027 (3)	0.029 (3)	−0.009 (2)	0.012 (3)	−0.007 (2)
C31	0.031 (3)	0.024 (3)	0.041 (4)	−0.013 (3)	0.007 (3)	−0.004 (3)
C32	0.030 (3)	0.042 (4)	0.033 (3)	−0.016 (3)	0.003 (3)	0.007 (3)
C33	0.026 (3)	0.044 (4)	0.022 (3)	−0.011 (3)	0.005 (3)	0.007 (3)
C34	0.025 (3)	0.032 (3)	0.018 (3)	−0.005 (2)	0.010 (2)	0.005 (2)
C20	0.031 (3)	0.023 (3)	0.016 (3)	−0.004 (2)	0.008 (2)	0.001 (2)
C21	0.036 (3)	0.028 (3)	0.024 (3)	−0.003 (3)	0.015 (3)	0.006 (2)
C22	0.038 (3)	0.024 (3)	0.029 (3)	−0.004 (3)	0.018 (3)	0.002 (2)
C23	0.040 (4)	0.029 (3)	0.035 (3)	−0.015 (3)	0.010 (3)	−0.004 (3)
C24	0.031 (3)	0.031 (3)	0.020 (3)	−0.010 (3)	0.003 (3)	−0.003 (2)
C25	0.079 (5)	0.038 (4)	0.026 (3)	−0.016 (4)	0.011 (4)	0.014 (3)
C26	0.101 (7)	0.043 (5)	0.046 (5)	−0.031 (5)	0.004 (5)	0.013 (4)
C27	0.060 (5)	0.060 (5)	0.066 (5)	−0.013 (4)	0.013 (4)	0.041 (5)
C28	0.034 (4)	0.080 (6)	0.051 (4)	0.021 (4)	0.026 (3)	0.047 (4)
C29	0.036 (3)	0.045 (4)	0.033 (3)	0.018 (3)	0.021 (3)	0.023 (3)
N1	0.018 (2)	0.015 (2)	0.0102 (18)	−0.0002 (16)	0.0081 (16)	−0.0008 (15)
N4	0.017 (2)	0.022 (2)	0.017 (2)	−0.0036 (18)	0.0053 (18)	−0.0004 (18)
N2	0.024 (2)	0.024 (2)	0.014 (2)	−0.0051 (19)	0.0081 (19)	−0.0025 (17)
N3	0.024 (2)	0.030 (3)	0.022 (2)	−0.001 (2)	0.0095 (19)	0.010 (2)
O1	0.0172 (16)	0.0210 (18)	0.0071 (14)	−0.0009 (14)	0.0062 (13)	−0.0020 (13)
O2	0.0154 (17)	0.024 (2)	0.0163 (17)	−0.0009 (14)	0.0078 (14)	0.0014 (14)
O3	0.0202 (17)	0.0175 (17)	0.0138 (16)	0.0008 (15)	0.0097 (14)	−0.0008 (14)
O16	0.0166 (17)	0.0129 (17)	0.0150 (16)	0.0017 (14)	0.0083 (14)	0.0001 (14)
O12	0.027 (2)	0.0153 (18)	0.0218 (19)	0.0031 (15)	0.0130 (16)	0.0024 (15)
O13	0.027 (2)	0.0151 (18)	0.0211 (19)	−0.0010 (15)	0.0110 (16)	−0.0016 (14)
O6	0.0217 (19)	0.028 (2)	0.0166 (18)	−0.0034 (16)	0.0097 (16)	−0.0021 (15)
O7	0.0196 (18)	0.0269 (19)	0.0148 (17)	−0.0044 (16)	0.0075 (14)	−0.0033 (15)
O4	0.029 (2)	0.0138 (18)	0.0187 (18)	−0.0010 (15)	0.0140 (16)	−0.0011 (14)
O5	0.0238 (19)	0.0136 (17)	0.0241 (19)	−0.0008 (15)	0.0128 (16)	0.0004 (14)
O10	0.0186 (18)	0.0202 (18)	0.0143 (17)	−0.0048 (14)	0.0069 (15)	−0.0021 (13)
O11	0.0226 (19)	0.0199 (19)	0.0113 (17)	−0.0067 (15)	0.0066 (15)	−0.0037 (14)
O8	0.0207 (19)	0.0213 (19)	0.0242 (19)	−0.0028 (15)	0.0135 (16)	−0.0108 (15)
O9	0.0197 (17)	0.0185 (18)	0.0225 (18)	−0.0017 (15)	0.0120 (15)	−0.0064 (15)
O15	0.028 (2)	0.0177 (19)	0.0164 (18)	−0.0035 (15)	0.0073 (16)	−0.0070 (14)
O14	0.0224 (19)	0.031 (2)	0.0153 (17)	−0.0058 (17)	0.0076 (15)	−0.0001 (15)
O17	0.062 (5)	0.106 (8)	0.076 (7)	0.042 (5)	0.043 (5)	0.068 (6)
O17A	0.047 (11)	0.094 (16)	0.048 (12)	−0.002 (11)	0.027 (9)	0.016 (11)

### Hexa-µ-acetato-chlorido(µ-N,2-dioxodobenzene-1-carboximidato)-µ3-oxido-tetrairon(III)–water (1/1) (1-Cl) Geometric parameters (Å, º)

**Table:**

Cl1—Fe1	2.2963 (14)	C14—O10	1.256 (6)
Fe2—O3	1.918 (3)	C14—O11	1.269 (6)
Fe2—O16	1.940 (3)	C14—C15	1.503 (7)
Fe2—O6	2.015 (4)	C15—H15A	0.9800
Fe2—O12	2.050 (3)	C15—H15B	0.9800
Fe2—O4	2.070 (3)	C15—H15C	0.9800
Fe2—N1	2.075 (4)	C12—O8	1.259 (6)
Fe4—O16	1.886 (3)	C12—O9	1.272 (6)
Fe4—O13	2.011 (3)	C12—C13	1.504 (7)
Fe4—O1	2.033 (3)	C13—H13A	0.9800
Fe4—O10	2.035 (3)	C13—H13B	0.9800
Fe4—O8	2.039 (3)	C13—H13C	0.9800
Fe4—O15	2.039 (3)	C18—O14	1.258 (6)
Fe1—O2	1.957 (3)	C18—O15	1.262 (6)
Fe1—O14	1.982 (3)	C18—C19	1.493 (7)
Fe1—O1	2.025 (3)	C19—H19A	0.9800
Fe1—N3	2.165 (5)	C19—H19B	0.9800
Fe1—N2	2.194 (4)	C19—H19C	0.9800
Fe3—O16	1.866 (3)	C30—N4	1.346 (7)
Fe3—O5	2.012 (3)	C30—C31	1.383 (8)
Fe3—O11	2.016 (3)	C30—H30	0.9500
Fe3—O9	2.023 (3)	C31—C32	1.382 (9)
Fe3—O7	2.030 (3)	C31—H31	0.9500
Fe3—N4	2.206 (4)	C32—C33	1.369 (9)
C1—O2	1.297 (6)	C32—H32	0.9500
C1—N1	1.306 (6)	C33—C34	1.378 (8)
C1—C2	1.485 (6)	C33—H33	0.9500
C2—C7	1.392 (7)	C34—N4	1.338 (6)
C2—C3	1.400 (6)	C34—H34	0.9500
C3—C4	1.370 (7)	C20—N2	1.338 (7)
C3—H3	0.9500	C20—C21	1.383 (8)
C4—C5	1.392 (8)	C20—H20	0.9500
C4—H4	0.9500	C21—C22	1.369 (8)
C5—C6	1.372 (7)	C21—H21	0.9500
C5—H5	0.9500	C22—C23	1.390 (8)
C6—C7	1.414 (6)	C22—H22	0.9500
C6—H6	0.9500	C23—C24	1.383 (8)
C7—O3	1.322 (6)	C23—H23	0.9500
C16—O12	1.254 (6)	C24—N2	1.343 (7)
C16—O13	1.260 (6)	C24—H24	0.9500
C16—C17	1.505 (7)	C25—N3	1.332 (8)
C17—H17A	0.9800	C25—C26	1.388 (9)
C17—H17B	0.9800	C25—H25	0.9500
C17—H17C	0.9800	C26—C27	1.367 (11)
C10—O7	1.257 (6)	C26—H26	0.9500
C10—O6	1.260 (6)	C27—C28	1.387 (12)
C10—C11	1.504 (8)	C27—H27	0.9500
C11—H11A	0.9800	C28—C29	1.370 (10)
C11—H11B	0.9800	C28—H28	0.9500
C11—H11C	0.9800	C29—N3	1.339 (7)
C8—O5	1.255 (6)	C29—H29	0.9500
C8—O4	1.259 (6)	N1—O1	1.416 (4)
C8—C9	1.502 (7)	O17—H17D	0.85 (3)
C9—H9A	0.9800	O17—H17E	0.84 (3)
C9—H9B	0.9800	O17A—H17F	0.86 (3)
C9—H9C	0.9800	O17A—H17G	0.85 (3)
			
O3—Fe2—O16	172.17 (14)	H9B—C9—H9C	109.5
O3—Fe2—O6	90.17 (14)	O10—C14—O11	124.7 (4)
O16—Fe2—O6	97.63 (13)	O10—C14—C15	118.1 (4)
O3—Fe2—O12	90.00 (14)	O11—C14—C15	117.2 (4)
O16—Fe2—O12	91.14 (14)	C14—C15—H15A	109.5
O6—Fe2—O12	86.91 (14)	C14—C15—H15B	109.5
O3—Fe2—O4	88.54 (14)	H15A—C15—H15B	109.5
O16—Fe2—O4	90.71 (13)	C14—C15—H15C	109.5
O6—Fe2—O4	90.10 (14)	H15A—C15—H15C	109.5
O12—Fe2—O4	176.67 (15)	H15B—C15—H15C	109.5
O3—Fe2—N1	85.84 (14)	O8—C12—O9	125.3 (5)
O16—Fe2—N1	86.39 (14)	O8—C12—C13	118.2 (5)
O6—Fe2—N1	175.53 (15)	O9—C12—C13	116.5 (4)
O12—Fe2—N1	91.08 (15)	C12—C13—H13A	109.5
O4—Fe2—N1	91.80 (15)	C12—C13—H13B	109.5
O16—Fe4—O13	95.04 (14)	H13A—C13—H13B	109.5
O16—Fe4—O1	88.71 (13)	C12—C13—H13C	109.5
O13—Fe4—O1	91.05 (14)	H13A—C13—H13C	109.5
O16—Fe4—O10	96.37 (13)	H13B—C13—H13C	109.5
O13—Fe4—O10	168.28 (14)	O14—C18—O15	125.1 (5)
O1—Fe4—O10	91.88 (13)	O14—C18—C19	117.0 (5)
O16—Fe4—O8	93.00 (13)	O15—C18—C19	117.9 (5)
O13—Fe4—O8	87.50 (14)	C18—C19—H19A	109.5
O1—Fe4—O8	177.85 (14)	C18—C19—H19B	109.5
O10—Fe4—O8	89.23 (14)	H19A—C19—H19B	109.5
O16—Fe4—O15	176.24 (14)	C18—C19—H19C	109.5
O13—Fe4—O15	86.51 (14)	H19A—C19—H19C	109.5
O1—Fe4—O15	87.84 (14)	H19B—C19—H19C	109.5
O10—Fe4—O15	82.26 (13)	N4—C30—C31	122.3 (5)
O8—Fe4—O15	90.48 (14)	N4—C30—H30	118.9
O2—Fe1—O14	167.24 (14)	C31—C30—H30	118.9
O2—Fe1—O1	77.46 (12)	C32—C31—C30	119.7 (6)
O14—Fe1—O1	90.32 (13)	C32—C31—H31	120.2
O2—Fe1—N3	94.22 (15)	C30—C31—H31	120.2
O14—Fe1—N3	88.66 (16)	C33—C32—C31	117.5 (6)
O1—Fe1—N3	86.72 (15)	C33—C32—H32	121.3
O2—Fe1—N2	89.67 (15)	C31—C32—H32	121.3
O14—Fe1—N2	86.85 (16)	C32—C33—C34	120.6 (6)
O1—Fe1—N2	90.99 (15)	C32—C33—H33	119.7
N3—Fe1—N2	174.96 (17)	C34—C33—H33	119.7
O2—Fe1—Cl1	93.87 (10)	N4—C34—C33	122.1 (5)
O14—Fe1—Cl1	98.56 (11)	N4—C34—H34	118.9
O1—Fe1—Cl1	170.45 (10)	C33—C34—H34	118.9
N3—Fe1—Cl1	89.97 (12)	N2—C20—C21	123.1 (5)
N2—Fe1—Cl1	92.98 (12)	N2—C20—H20	118.5
O16—Fe3—O5	93.99 (14)	C21—C20—H20	118.5
O16—Fe3—O11	98.53 (13)	C22—C21—C20	119.0 (5)
O5—Fe3—O11	89.69 (14)	C22—C21—H21	120.5
O16—Fe3—O9	93.46 (14)	C20—C21—H21	120.5
O5—Fe3—O9	172.54 (14)	C21—C22—C23	118.8 (5)
O11—Fe3—O9	88.91 (14)	C21—C22—H22	120.6
O16—Fe3—O7	97.10 (14)	C23—C22—H22	120.6
O5—Fe3—O7	89.76 (14)	C24—C23—C22	119.0 (5)
O11—Fe3—O7	164.36 (14)	C24—C23—H23	120.5
O9—Fe3—O7	89.61 (14)	C22—C23—H23	120.5
O16—Fe3—N4	178.69 (15)	N2—C24—C23	122.3 (5)
O5—Fe3—N4	84.80 (15)	N2—C24—H24	118.8
O11—Fe3—N4	81.96 (14)	C23—C24—H24	118.8
O9—Fe3—N4	87.76 (15)	N3—C25—C26	123.5 (7)
O7—Fe3—N4	82.43 (15)	N3—C25—H25	118.2
O2—C1—N1	120.8 (4)	C26—C25—H25	118.2
O2—C1—C2	118.6 (4)	C27—C26—C25	118.8 (8)
N1—C1—C2	120.6 (4)	C27—C26—H26	120.6
C7—C2—C3	120.3 (4)	C25—C26—H26	120.6
C7—C2—C1	123.3 (4)	C26—C27—C28	118.5 (7)
C3—C2—C1	116.2 (4)	C26—C27—H27	120.8
C4—C3—C2	121.3 (5)	C28—C27—H27	120.8
C4—C3—H3	119.4	C29—C28—C27	118.9 (6)
C2—C3—H3	119.4	C29—C28—H28	120.6
C3—C4—C5	118.8 (5)	C27—C28—H28	120.6
C3—C4—H4	120.6	N3—C29—C28	123.6 (7)
C5—C4—H4	120.6	N3—C29—H29	118.2
C6—C5—C4	120.9 (5)	C28—C29—H29	118.2
C6—C5—H5	119.5	C1—N1—O1	111.2 (4)
C4—C5—H5	119.5	C1—N1—Fe2	129.6 (3)
C5—C6—C7	120.9 (5)	O1—N1—Fe2	118.6 (2)
C5—C6—H6	119.5	C34—N4—C30	117.8 (5)
C7—C6—H6	119.5	C34—N4—Fe3	121.4 (4)
O3—C7—C2	124.2 (4)	C30—N4—Fe3	120.6 (4)
O3—C7—C6	118.0 (4)	C20—N2—C24	117.8 (5)
C2—C7—C6	117.7 (4)	C20—N2—Fe1	121.1 (3)
O12—C16—O13	125.6 (4)	C24—N2—Fe1	121.0 (4)
O12—C16—C17	117.4 (5)	C25—N3—C29	116.7 (5)
O13—C16—C17	117.0 (4)	C25—N3—Fe1	121.5 (4)
C16—C17—H17A	109.5	C29—N3—Fe1	121.8 (4)
C16—C17—H17B	109.5	N1—O1—Fe1	114.1 (2)
H17A—C17—H17B	109.5	N1—O1—Fe4	114.4 (2)
C16—C17—H17C	109.5	Fe1—O1—Fe4	131.50 (15)
H17A—C17—H17C	109.5	C1—O2—Fe1	116.4 (3)
H17B—C17—H17C	109.5	C7—O3—Fe2	132.3 (3)
O7—C10—O6	125.7 (5)	Fe3—O16—Fe4	120.97 (16)
O7—C10—C11	116.3 (5)	Fe3—O16—Fe2	121.56 (17)
O6—C10—C11	117.9 (5)	Fe4—O16—Fe2	117.36 (16)
C10—C11—H11A	109.5	C16—O12—Fe2	131.3 (3)
C10—C11—H11B	109.5	C16—O13—Fe4	131.9 (3)
H11A—C11—H11B	109.5	C10—O6—Fe2	131.0 (3)
C10—C11—H11C	109.5	C10—O7—Fe3	134.1 (3)
H11A—C11—H11C	109.5	C8—O4—Fe2	133.5 (3)
H11B—C11—H11C	109.5	C8—O5—Fe3	131.1 (3)
O5—C8—O4	126.0 (4)	C14—O10—Fe4	128.4 (3)
O5—C8—C9	116.9 (5)	C14—O11—Fe3	133.1 (3)
O4—C8—C9	117.1 (4)	C12—O8—Fe4	132.8 (3)
C8—C9—H9A	109.5	C12—O9—Fe3	127.4 (3)
C8—C9—H9B	109.5	C18—O15—Fe4	130.4 (3)
H9A—C9—H9B	109.5	C18—O14—Fe1	134.5 (3)
C8—C9—H9C	109.5	H17D—O17—H17E	114 (7)
H9A—C9—H9C	109.5	H17F—O17A—H17G	104 (7)

### Hexa-µ-acetato-chlorido(µ-N,2-dioxodobenzene-1-carboximidato)-µ3-oxido-tetrairon(III)–water (1/1) (1-Cl) Hydrogen-bond geometry (Å, º)

**Table:**

*D*—H···*A*	*D*—H	H···*A*	*D*···*A*	*D*—H···*A*
C6—H6···O17*A*^i^	0.95	2.64	3.486 (19)	149
C15—H15*C*···O15^ii^	0.98	2.62	3.581 (7)	166
C19—H19*A*···O11^iii^	0.98	2.61	3.424 (7)	141
C20—H20···N1	0.95	2.65	3.429 (7)	140
C20—H20···O1	0.95	2.53	3.100 (6)	118
C21—H21···O4^iv^	0.95	2.56	3.403 (6)	148
C23—H23···Cl1^v^	0.95	2.97	3.697 (6)	135
C24—H24···Cl1	0.95	2.75	3.301 (6)	118
C25—H25···O2	0.95	2.55	3.126 (7)	119
C26—H26···O17^ii^	0.95	2.20	3.014 (12)	144
C26—H26···O17*A*^ii^	0.95	2.60	3.17 (3)	118
C29—H29···O14	0.95	2.48	3.007 (7)	115
O17—H17*D*···Cl1^vi^	0.85 (3)	2.80 (3)	3.647 (9)	174 (15)
O17—H17*E*···O3^vii^	0.84 (3)	2.03 (5)	2.830 (8)	159 (12)

Symmetry codes: (i) *x*, *y*, *z*+1; (ii) −*x*, *y*+1/2, −*z*+1; (iii) −*x*, *y*−1/2, −*z*+1; (iv) −*x*, *y*−1/2, −*z*+2; (v) −*x*−1, *y*−1/2, −*z*+1; (vi) *x*+1, *y*, *z*; (vii) *x*, *y*, *z*−1.
